# Precision ultrasound sensing on a chip

**DOI:** 10.1038/s41467-018-08038-4

**Published:** 2019-01-10

**Authors:** Sahar Basiri-Esfahani, Ardalan Armin, Stefan Forstner, Warwick P. Bowen

**Affiliations:** 1 ARC Centre for Engineered Quantum Systems, School of Mathematics and Physics, The University of Queensland, St. Lucia, QLD 4072 Australia; 2 Department of Physics, Swansea University, Singleton Park, Swansea, SA2 8PP Wales UK

## Abstract

Ultrasound sensors have wide applications across science and technology. However, improved sensitivity is required for both miniaturisation and increased spatial resolution. Here, we introduce cavity optomechanical ultrasound sensing, where dual optical and mechanical resonances enhance the ultrasound signal. We achieve noise equivalent pressures of 8–300 μPa H*z*^−1/2^ at kilohertz to megahertz frequencies in a microscale silicon-chip-based sensor with >120 dB dynamic range. The sensitivity far exceeds similar sensors that use an optical resonance alone and, normalised to the sensing area, surpasses previous air-coupled ultrasound sensors by several orders of magnitude. The noise floor is dominated by collisions from molecules in the gas within which the acoustic wave propagates. This approach to acoustic sensing could find applications ranging from biomedical diagnostics, to autonomous navigation, trace gas sensing, and scientific exploration of the metabolism-induced-vibrations of single cells.

## Abstract (web summary)

With the wide adoption of ultrasound methods in biomedical and technological diagnostics, sensitive probes are in demand. Here, the authors employ cavity optomechanics where optical and mechanical resonances are coupled, both enhancing the sensitivity of the device and allowing its chip-integration.

## Introduction

Over the past decade, cavity optomechanical sensors have emerged as a new class of ultraprecise photonic sensors^1–5^. These sensors integrate a high-quality mechanical resonator with a high-quality optical cavity (for e.g., see^6^). The mechanical resonator amplifies the mechanical vibrations introduced by resonant signals and provides isolation from environmental thermal noise, while the cavity resonantly enhances the optical response to the mechanical vibrations. A characteristic feature of cavity optomechanical sensors is that they are often only limited by optical shot noise and mechanical thermal noise, allowing the intrinsic limits in sensing performance to be approached^7^. This provides the ability to perform exquisitely sensitive optical measurements, with sub-attometre precision^8^. At kilometre scales it has proved crucial for the successful detection of gravitational waves^9^; while at micro- and nano-scales it has enabled high-performance acceleration, single-molecule, temperature and magnetic field sensing^1–3,10–13^, as well as provided a new approach to control the quantum physics of massive objects, allowing quantum ground-state cooling^14–16^ and the generation of macroscopic non-classical states of motion^17,18^, with applications in future quantum technologies (for e.g., see^19–21^).

Detection of acoustic waves is essential for many applications including medical diagnostics, sonar, navigation, trace gas sensing and industrial processes^22,23^. Most acoustic sensors transform an acoustic pressure wave into vibrations of a mechanical element, and detect these vibrations electrically via changes in piezoelectricity^24^, resistivity^25^, magnetic transduction or capacitance^26^. For many applications, high spatial, temporal and directional resolution is a key requirement. This has driven development towards both ultrasonic frequencies, with their correspondingly short-acoustic wavelengths, and microscale sensing devices that are capable of resolving such waves at, near, or beyond their diffraction limit^27^. The degradation in acoustic sensitivity that comes hand-in-hand with operation at higher frequencies and with smaller sensing areas presents a major challenge^28^. While, for an acoustic wave propagating through gas, the sensitivity is only fundamentally limited by the random momentum kicks from gas molecules as they collide with the sensor, all existing acoustic sensors are far from this limit. Their noise floor is, instead, typically dominated by electronic noise. This has motivated recent progress in photonic acoustic sensors^29–31^.

In this article we extend cavity optomechanical sensing to the measurement of acoustic and ultrasonic waves, using a lithographically fabricated device suspended above a silicon chip via thin tethers. By engineering its structure for high-acoustic sensitivity, we reach the regime where gas molecule collisions dominate the noise floor. This allows noise equivalent pressures of 8–300 μPa Hz^−1/2^ at a range of frequencies between 1 kHz and 1 MHz. Compared to acoustic sensors that use similar, but non-suspended, optical cavities and rely on refractive index shifts and static deformations rather than nanomechanical resonances^32^, the peak sensitivity represents a more than three order-of-magnitude advance. Normalised by device area, it outperforms all previous air-coupled ultrasound sensors by two orders-of-magnitude at ultrasound frequencies from 80 kHz to 1 MHz.

## Results

### Cavity optomechanical acoustic sensing

**Fig. 1:**
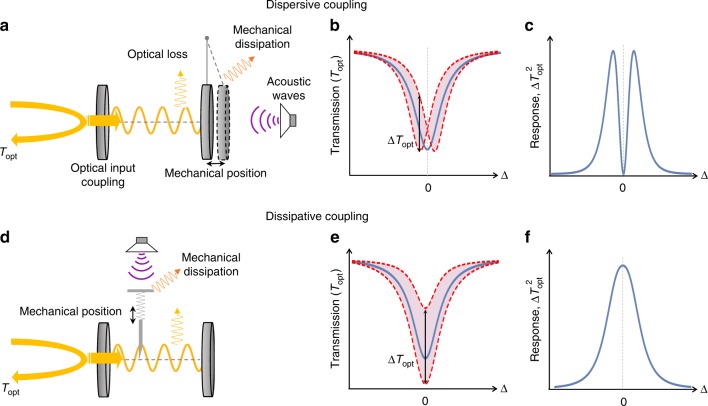
Principles of dispersive and dissipative cavity optomechanical acoustic sensing. **a**, **d** Conceptual schematics of Fabry–Pérot cavity-based dispersive (**a**) and dissipative (**d**) sensors. In **a** an applied acoustic force drives harmonic oscillation of a movable cavity end-mirror modulating the length and resonance frequency of the cavity. In **d** the force drives a mechanical element that modulates the decay rate of the cavity. The modulation is monitored via the change in optical transmission (*T*_opt_) from the cavity. **b**, **e** Cavity transmission in the presence of dispersive and dissipative coupling, respectively. The solid blue curves show the cavity transmission for the initial position of the mechanical element while the red dashed curves show the modified cavity transmission due to displacement of the mechanical element. **c**, **f** Amplitude of external-force driven modulation in transmission (Δ*T*_opt_) of the cavity optomechanical system for dispersive and dissipative coupling, respectively, versus the detuning Δ of the input laser field from the cavity resonance

In general, cavity optomechanical sensors consist of a mechanically compliant element coupled to an optical cavity. The mechanical element is displaced in response to an external stimulus—in our case an acoustic wave. The optical cavity resonantly enhances the optical response to this displacement, allowing precise measurement of the stimulus. Commonly, the coupling from displacement to optical response can occur in one of two ways: dispersive^33^ or dissipative coupling^34–36^, both of which are used in our sensor. With dispersive coupling, the mechanical displacement alters the cavity length, and therefore optical resonance frequency (See Fig. 1a, b). In dissipative coupling, the displacement instead alters the cavity decay rate, by modifying either the optical input coupling or intracavity loss (see Fig. 1d, e). The concept of ultrasound sensors based on each coupling mechanism is shown in Fig. 1, using a Fabry–Pérot cavity as an illustrative example. In both cases, the output optical signal is linearly proportional to the amplitude of the applied acoustic wave.

In the simple case where the mechanical element has a single mechanical resonance, the minimum detectable acoustic pressure for both dispersive and dissipative cavity optomechanical sensing is given by

1 $$P_{{\mathrm{min}}}(\omega ) = \frac{1}{{r\zeta A}}\sqrt {2\left( {\mu l + m\gamma } \right)k_BT + \frac{1}{{N\left| {\chi (\omega ,{\mathrm{\Delta }})} \right|^2}}} ,$$

where *A* and *T* are the area and temperature of the sensor, respectively, and we assume that the laser used to probe the optical response is shot noise limited (see Supplementary Note 1 for derivation). The acoustic pressure wave will only exert a force if it induces a pressure difference between the top and bottom surfaces of the mechanical element. This is quantified by *r*, the ratio of the pressure difference to the peak pressure at the antinode of the acoustic wave. *ζ* is the spatial overlap of the displacement profile of the mechanical sensing element with the incident pressure wave (see Supplementary Note 2). The first of the three terms under the square-root quantifies the thermomechanical noise introduced by collisions with molecules in the gas surrounding the resonator, where *μ* is the coefficient of viscosity of the gas and *l* is a device geometry-dependent characteristic length-scale. The second term quantifies the thermomechanical noise introduced by the fluctuation–dissipation theorem due to the intrinsic damping of the mechanical resonator. *m* is the resonator effective mass which is generally close to but less than the actual mass, and *γ* is the intrinsic mechanical damping rate. The third term quantifies the optical measurement noise, with *N* being the number of photons in the cavity and *χ*(*ω*, Δ) an optomechanical susceptibility which accounts for the optical and mechanical response of the sensor as a function of acoustic drive frequency *ω* and cavity detuning Δ. In the case relevant to our experiments, where the cavity decay rate is much faster than the acoustic drive frequency, the acoustic frequency dependence is determined solely by the mechanical response and is independent of coupling mechanism. On the other hand, the detuning dependence is fundamentally different for dispersive and dissipative coupling (see Supplementary Note 1). For optical intensity measurement, |*χ*| is zero when the probe laser is tuned to the cavity resonance (Δ = 0), and maximised when it is detuned by $\left| {\mathrm{\Delta }} \right| = \frac{\kappa }{{2\sqrt 3 }}$. Conversely, for dissipative coupling, |*χ*| is generally maximised for on-resonance optical driving. This difference is illustrated in Fig. 1b, e. We finally note that, while derived here for cavity optomechanical sensing, Eq. (1) is applicable quite generally when a mechanical resonance is used to enhance the response of an acoustic sensor in a gaseous environment (such as^24–26,37^)—only the measurement noise term need be replaced to align with the specific choice of transduction mechanism.

Fundamentally, the sensitivity of photoacoustic sensing is limited by the thermal energy of the medium through which the acoustic wave propagates. In liquids, resonant ultrasound sensors approach to within a factor of two of this thermal limit^38^. However, the far lower acoustic impedance of gaseous media greatly reduces both the magnitude of the thermal noise and the efficiency with which acoustic signals can be detected, significantly increasing the challenge^32^. In this case, the thermal limit results from collisions of gas molecules with the sensor surface, which introduces gas damping of the mechanical energy and is associated with the first term under the square-root in Eq. (1). For the characteristic viscous length-scale of our devices (*l* ~ 8 mm, see Supplementary Note 3), their area of *A* ~ 0.05 mm^2^, an ideal pressure participation ratio and spatial overlap (*r* = *ζ* = 1), and a surrounding gas of air at room temperature (*μ* = 1.8 × 10^−5^ kg m^−1^ s^−1^) we find this gas-damping thermal limit to be *P*_min_ ~ 1 μPa Hz^−1/2^. This predicted fundamental-noise limited sensitivity is many orders of magnitude superior to previously reported ultrasound sensors of comparable size^31^. For larger centimetre-scale sensors, the limit drops to tens of nanopascal levels, also well beyond the state-of-the-art. To reach it, the intrinsic mechanical damping rate (*γ*) must be smaller than the gas-damping rate (*γ*_gas_ = *μl*/*m*), such that a high quality, low mass, mechanical resonator is advantageous. Furthermore, the measurement noise must be small enough to allow resolution of the random thermal force from collisions of gas molecules with the resonator. In general, it has proved challenging to simultaneously satisfy these requirements. However, they align closely with the characteristics of optomechanical devices developed over the past decade to study the quantum physics of nanoscale motion (see e.g.,^39,40^).

### Sensor design and characterisation

**Fig. 2:**
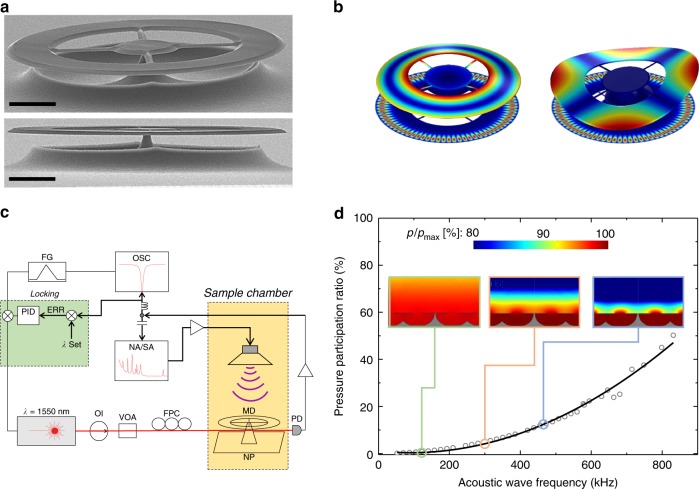
Device architecture and experimental schematic. **a** Scanning electron micrograph of a similar microdisk to that used in this study. The microdisk is an optical cavity which is evanescently coupled to a tapered optical fibre. The scale bar corresponds to 20 μm. **b** Finite-element simulations of the modeshapes of two typical mechanical modes of the microdisk (left: second-order flapping mode, right: crown mode). **c** The phase sensitive and thermally stabilised experimental setup used to characterise the sensor. NP nanopositioner, MD microdisk, PD photodetector, FPC fibre polarization controller, VOA variable optical attenuator, OI optical isolator, FG function generator, OSC digital oscilloscope, NA network analyser, SA spectrum analyser. **d** The simulated pressure participation ratio, i.e., the fraction of the total acoustic pressure acting on the mechanical structure, for a number of frequencies. The insets display the pressure distribution at 105, 281 and 421 kHz, respectively; while *p*/*p*_max_ is the ratio of the pressure to the pressure at the antinodes of the acoustic wave

Here, we develop a suspended spoked silica microdisk optomechanical system purpose-designed for ultrasensitive ultrasound detection, as shown in Fig. 2a. Similar to a regular microdisk cavity, light is confined in a high-quality whispering-gallery mode around the periphery of the disk, maximising both the optomechanical susceptibility *χ* and the intracavity photon number *N* for a given incident optical power. The use of thin spokes to suspend the disk above a silicon substrate both further increases the optomechanical susceptibility by increasing the compliance of the mechanical structure, and isolates the mechanical resonances, greatly suppressing the intrinsic mechanical damping^39^. One compromise associated with the use of spokes is a reduction in active sensing area. Here, we optimise the active area within the constraints of the device footprint to functionalise spoked microdisks for efficient ultrasound detection. We find that high-mechanical compliance and isolation can both be achieved while maintaining a 70% active area, such that the reduction in area only minimally influences the acoustic sensitivity.

While suspension of the mechanical element offers significant advantages in terms of mechanical quality and compliance, a potential disadvantage is that its underside is not isolated from the acoustic pressure wave. One might expect this to reduce the pressure difference across the resonator, decreasing the pressure participation ratio and degrading the acoustic sensitivity. To explore this behaviour, we perform finite-element simulations of an acoustic plane wave incident on a spoked silica microdisk, with results shown in Fig. 2d. The participation ratio is found to increase roughly quadratically with acoustic wave frequency, exceeding 50% at frequencies above 800 kHz. We attribute the quadratic dependence firstly to the increasing spatial gradient of the pressure wave with increasing frequency and, secondly, to an increasing resonant confinement of sound between the sensor and the substrate, as the acoustic wavelength becomes comparable to the height of the airgap beneath the sensor.

The spoked microdisk is photolithographically fabricated with outer and inner radii of 148 and 82 μm, respectively, and *a* ~ 1.8 μm device thickness, resulting in a small mass of approximately 230 ng (see Methods and SEM image in Fig. 2a). The probe laser is evanescently coupled into, and out of, the microdisk via an optical nanofibre, facilitating direct coupling into fibre-optic systems. We note that on-chip packaging is also possible by replacing the nanofibre with an integrated optical waveguide^41^. The microdisk supports families of mechanical eigenmodes that can be resonantly driven via an acoustic field (see Fig. 2b). The dominant effect of microdisk vibrations on the cavity resonance is generally to modify the resonance frequency, providing a mechanism for dispersive optomechanical sensing. However, vibrations can also enable dissipative sensing, modifying the distance between fibre and microdisk and therefore the cavity input coupling.

Using the experimental setup shown in Fig. 2c, the mechanical and optical modes of the sensor, as well as its acoustic response, were investigated via their effect on the transmission of the probe laser through the nanofibre. An optical cavity mode with wavelength of around *λ* = 1555.7 nm, in the telecommunications C-band, and with intrinsic quality factor of 3.6 × 10^6^ was selected for the experiments (see Methods and Supplementary Fig. 3). A feedback loop was used to lock the laser wavelength at a fixed detuning with respect to this mode, such that the experiment was insensitive to low-frequency thermal fluctuations in the cavity and optical fibre circuit and drift of the probe laser wavelength.

**Fig. 3:**
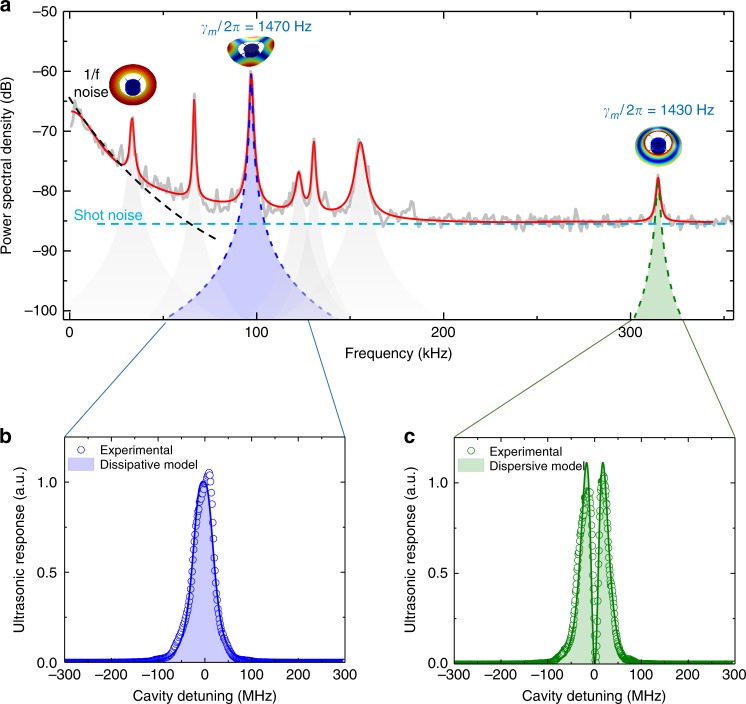
Noise spectrum and coupling mechanisms. **a** The noise spectral density of the microdisk coupled to the tapered fibre in the absence of acoustic signal. The blue dashed line specifies the shot noise level given by the laser intensity, and the black dashed line corresponds to the 1/*f* noise. The shaded Lorentzian peaks specify the combined noise due to intrinsic damping and gas damping for several mechanical modes of the device. The green and blue shading highlights examples of dispersively and dissipatively coupled mechanical modes, respectively. *γ*_*m*_ quantifies the total mechanical dissipation rate of each of these modes, including both gas and intrinsic damping. **b**, **c** The ultrasonic response as a function of laser-cavity detuning at frequencies of 98 and 315 kHz, resonant with the second-order crown and flapping modes of the disk, respectively. The shaded areas are fits based upon the theoretical expectation for system response as function of detuning (see Supplementary Fig. 1) corresponding to dissipative (**b**) and dispersive (**c**) coupling, respectively

To investigate the response, noise performance and sensitivity of the sensor, we detuned the laser away from the optical resonance to the point of maximum slope with respect to the cavity dispersion, optimising the dispersive transduction of acoustic signals. The noise spectral density of the sensor was then measured using a spectrum analyser, as shown in Fig. 3. At low frequencies (≲50 kHz), the dominant noise mechanism is 1/*f* noise. At higher frequencies, the noise floor is dominated either by laser shot noise or, near the resonance frequencies of mechanical eigenmodes, thermomechanical noise due to the combination of intrinsic and gas damping with characteristic sharply peaked Lorentzian frequency response.

In order to quantitatively verify our model for cavity optomechanical acoustic sensing (see Eq. (1) and Fig. 1c, f), we examined the acoustic response for the second-order crown and flapping modes of the microdisk shown in Fig. 2b. A piezo-electric element (PZT) was used as an ultrasonic transmitter, creating an ultrasonic wave at each frequency, and the response of the sensor was analysed using a vector network analyser. Specifically, the off-diagonal scattering parameter (i.e., the coherent power transmission from the PZT to the photodetector through the sensor) was recorded as a function of laser-cavity detuning. The results are shown in the bottom panels of Fig. 3. The response of the flapping mode is zero on cavity resonance, with maxima on either side, characteristic of the usual dispersive coupling (c.f. Fig. 1f). On the other hand, the crown mode features a maximum at zero detuning, characteristic of dissipative coupling (c.f. Fig. 1c). Dissipative coupling has been observed previously in a waveguide-coupled microdisk^36^. In our case it is most likely due to the large vertical displacement amplitude of the mode which modulates the taper-microcavity separation, combined with first-order suppression of dispersive coupling inherent to crown modes. The results show very good agreement to respective fits to dissipative and dispersive coupling, as shown in Fig. 3, validating the theoretical models for both sensing mechanisms.

### Characterising dynamic range and sensitivity of the sensor

**Fig. 4:**
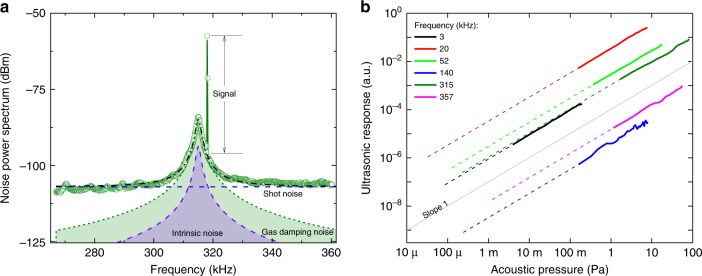
Evaluation of the noise equivalent pressure sensitivity and the linear dynamic range. **a** Noise spectral density of the sensor near a mechanical mode of the microdisk measured at an electrical bandwidth of 200 Hz. An ultrasonic pressure of 120 mPa at frequency of 318 kHz is applied to the device resulting in a signal-to-noise ratio of ~40 dB. The shot noise is shown with the dashed blue line. The thermomechanical noise introduced by intrinsic and gas damping is shown, respectively, by the purple and green shaded Lorenztian’s. The total noise is fitted with the black dash-dot line, in good agreement with theory, and is dominated by gas damping noise between 306 and 325 kHz. **b** Ultrasonic response of the sensor at different frequencies as a function of ultrasonic pressure. The dashed grey line is a guide to the eye indicating the expected slope for a linear response. The linear dynamic range (LDR) is >120 dB for a measurement integration time of 1 s, with its upper limit dictated by the measurement setup rather than the acoustic response. The solid lines correspond to the measured data for each frequency and the dashed lines connect these to the noise equivalent pressure that sets the lower limit of the LDR

To experimentally quantify the noise equivalent pressure of the sensor, we interferometrically calibrated the displacement of the PZT element as function of its drive frequency. The acoustic pressure generated by the PZT was calculated from its displacement, air acoustic impedance and its distance to the sensor (see Supplementary Note 5). The ultrasonic response of the system was then measured at different frequencies for which the applied pressure was known. Figure 4a shows, as an example, the response at 318 kHz in the wing of the second-order flapping mode, relative to both the shot noise and thermomechanical noise introduced by intrinsic and gas damping. The signal-to-noise ratio (SNR) ~ 40 dB with an applied pressure of *P*_applied_ = 120 mPa, measured over an integration time of *τ* = Δ*f*^−1^, where Δ*f* = 200 Hz is the spectrum analyser resolution bandwidth. The noise equivalent pressure can then be calculated as

2 $$P_{{\mathrm{min}}}(\omega ) = \sqrt {\frac{\tau }{{{\mathrm{SNR}}}}} \times P_{{\mathrm{applied}}}(\omega )\sim 84\,\mu {\rm{Pa}}\,{\mathrm{Hz}}^{ - 1/2}.$$

This is in reasonably good agreement with the thermomechanical noise-dominated noise equivalent pressure predicted from Eq. (1) of 100 μPa Hz^−1/2^, given the device area and temperature (*T* = 300 K), our simulated pressure participation ratio at 318 kHz of *r* = 0.055, the effective mass of the flapping mode of *m* = 110 ng, its measured total mechanical damping rate *γ*_m_/2*π* = (*γ* + *γ*_gas_)/2*π* = 1430 Hz, and its spatial overlap *ζ* = 0.14 with a plane pressure wave (see Supplementary Note 2).

It is informative to examine the contributions to the noise equivalent pressure from intrinsic mechanical dissipation, optical shot noise and fundamental gas damping. As can be seen from Fig. 4a, at the second-order flapping mode resonance frequency the shot noise power spectrum is 13 dB below the combined thermomechanical noise from gas and intrinsic damping, contributing 5% in power to the total noise. The fluctuation–dissipation theorem dictates that the ratio of noise power introduced by gas damping and intrinsic mechanical damping is equal to the ratio of the damping rates, as may be directly confirmed from Eq. (1). By measuring the mechanical damping rate of the flapping mode as a function of background pressure, we isolated these two components, finding that *γ*_gas_/2*π* = 1,260 Hz and *γ*/2*π* = 170 Hz (see Supplementary Fig. 2). The gas damping noise power therefore dominates by a factor of *γ*_gas_/*γ* ~ 7.4. All-in-all, these results show that, at this acoustic frequency, the noise equivalent pressure of the sensor is within 9% of the noise floor introduced by thermal collisions of gas molecules with the sensing element. This gas damping noise floor is fundamental, in that it cannot be eliminated without removing the gas through which the acoustic wave itself propagates. To our knowledge, our sensor is the first acoustic sensor which is sufficiently sensitive for it to dominate.

The resonantly enhanced bandwidth of the sensor around the second-order flapping mode is given by the frequency band where the combined thermomechanical noise dominates shot noise, i.e., between 306 and 325 kHz. The noise equivalent pressure is relatively constant over this frequency range, before degrading at frequencies further from resonance. This bandwidth could, in future, be extended by increasing the optical power used to probe the sensor (and therefore *N* in Eq. (1)) or even by using quantum correlations to reduce the optical noise level for fixed optical power^42^.

To explore the wider bandwidth, we measured the response and noise equivalent pressure for acoustic waves over the frequency range from 1 kHz to 1 MHz (see Supplementary Figs. 6 and 7). As expected for a resonant sensor, both parameters vary significantly over this range, exhibiting sharp resonant features. Resonantly enhanced narrowband sensitivities of 8–300 μPa Hz^−1/2^ are achieved for many frequencies across the range, with a broadband sensitivity better than 10 mPa Hz^−1/2^ maintained at all measured frequencies. The upper limit of 1 MHz is not intrinsic, but rather introduced by the inability to generate acoustic waves at higher frequencies due to the frequency response of our PZT transducer and the high acoustic attenuation of air at high frequencies. Indeed, mechanical resonances at hundred megahertz frequencies have been observed in cavity optomechanical systems of similar size to those reported here (see e.g.,^8^); while gigahertz resonance frequencies are available in smaller devices (see e.g.,^43^). Consequently, our approach can be expected to perform well into this higher frequency range. While the device was not optimised for audio frequencies, its performance at these lower frequencies remained sufficient to record a song in the lab environment by digitizing the output of the photo-detector with no further processing and filtering.

To investigate how the ultrasonic response changes when varying the magnitude of the acoustic pressure, we recorded the system response at various frequencies as a function of the applied pressure. As shown in Fig. 4b, the sensor has a linear dynamic range (LDR) of 120 dB. The lower bound on the LDR of any sensor is given by the noise equivalent signal (pressure sensitivity in case of an acoustic sensor) and the upper bound is the deviation point from linearity^44^. In our experiments, this upper limit is set by the maximum accessible pressure of ~100 Pa, with the sensor response linear throughout the range at all tested frequencies. Hence, the reported LDR is an underestimation.

## Discussion

It is interesting to compare the sensitivity of our sensor with existing ultrasound sensors. The peak sensitivity represents a more than three order-of-magnitude advance on previous comparable air-coupled optical sensors^13^, and is competitive with the best liquid-coupled piezoelectric sensors^38^ which benefit from four orders-of-magnitude larger sensing area and near-ideal acoustic impedance matching.

**Fig. 5:**
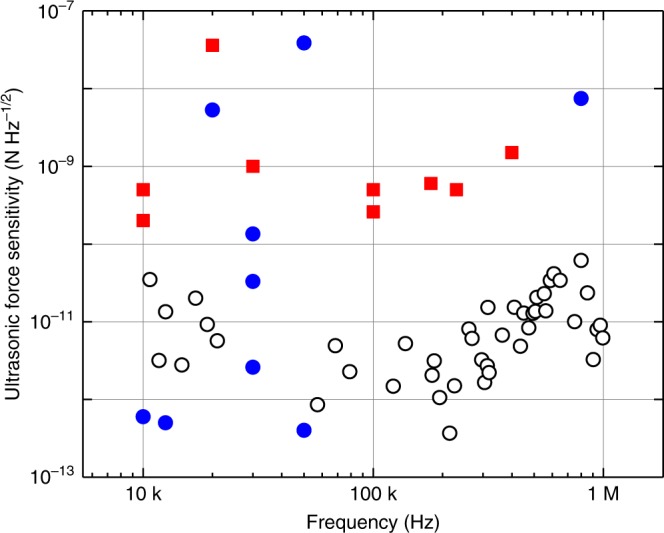
Ultrasonic force sensitivity in comparison with other air-coupled sensors. Ultrasonic force sensitivity is evaluated as the noise equivalent pressure sensitivity multiplied by the sensing area and plotted versus frequency: open circles correspond to this work and solid symbols show results of other optical (blue circles) and electrical (red squares) approaches. The improvement of the sensitivity in this work is especially notable between 80 kHz and 1 MHz. Citations to previous work are provided in the Supplementary Fig. 8

The force experienced by an ultrasound sensor scales linearly with sensing area. Consequently, as a general rule, sensitivity improves as the sensing area increases. To compare our sensor to ultrasound sensors of different sizes, we therefore calculate the ultrasonic force sensitivity, normalising the pressure sensitivity to area. Figure 5 shows the comparison to other air-coupled sensors over the frequency range from 10 kHz to 1 MHz. The performance is particularly good at frequencies between 80 kHz and 1 MHz, where the ultrasonic force sensitivity represents an advance of approximately two orders-of-magnitude. While this demonstrates that the sensor is an especially good acoustic force sensor, it is worth noting that while the absolute pressure sensitivity in Eq. (1) does include an explicit inverse-area scaling, it also includes implicit dependence on area through other parameters, such as the sensor mass and characteristic length-scale *l*. Consequently, the comparison between sensors of different size cannot be straightforwardly extended to absolute sensitivity.

Compared to liquid-coupled sensors, the peak ultrasonic force sensitivity of 370 fN Hz^−1/2^ is more than three-orders-of-magnitude superior to state-of-the-art piezoelectric sensors^29^, while also offering somewhat improved broadband sensitivity. The peak force sensitivity also exceeds optical liquid-coupled sensors, such as the Fabry–Pérot sensor in ref. ^30^, which has sensitivity of around 1.8 nN Hz^−1/2^ (see Supplementary Note 7) and microring sensors operating at 1.8 pN Hz^−1/2^^29^. That the sensor is comparable, both in terms of absolute pressure sensitivity and force sensitivity, with liquid-coupled sensors is notable given the large reduction in acoustic energy transport at the air-sensor interface due to the more than three orders-of-magnitude lower acoustic impedance of air compared with liquids^13^.

The sensor could be scaled straightforwardly to larger or smaller sizes, for improved absolute pressure sensitivity or improved resolution/high-frequency sensitivity, respectively. The sensitivity could be further improved by engineering the physical structure of the device to increase the pressure participation ratio, decrease the noise contribution from collisions with thermal gas molecules, and improve the overlap of the mechanical motion with the incident pressure wave. The participation ratio can be optimised, for a given acoustic wave frequency, by controlling the height of the sensor above the silicon substrate. Indeed, our modelling suggests that participation ratios even exceeding *r* = 1 are achievable due to resonant enhancement of the pressure wave between the substrate and device. This, in effect, would represent a microscale acoustic resonator fabricated on a silicon chip, with significant advantages over the bulk-machined acoustic resonators often used to enhance acoustic pressure waves in other approaches^22^. The overlap *ζ* could be increased to near unity by engineering the resonance frequency of a suitable mechanical mode, such as the first-order flapping mode, to coincide with the frequency of the pressure wave. The noise contribution from thermal gas molecule collisions is determined by the geometry-dependent characteristic length-scale *l*, which includes the effects of both squeeze-film molecular damping and air-drag damping. Squeeze-film damping arises from the gas trapped between the device and the substrate, and scales as inverse-height cubed. We estimate that it dominates air-drag damping by a factor of twenty for our current device design (see Supplementary Note 3), degrading the gas-damping-limited sensitivity by around a factor of five. By increasing the separation of the device from the substrate to both suppress squeeze-film damping and enhance the participation ratio, the sensitivity could be improved by more than a factor of one hundred, reaching the sub-micropascal regime.

The improved ultrasound sensitivity and microscale resolution offered by our new acoustic sensing technique has prospects for a range of applications. For instance, it could allow improved navigation and spatial imaging in unmanned and autonomous vehicles^45^; and higher sensitivity high-resolution photoacoustic trace gas sensing^22^. In trace gas sensing, the sensitivity reported here could allow detection of carbon dioxide at ten-part-per-billion concentrations with unprecedented spatial resolution (See Supplementary Note 8). This could, for example, enable measurements of the respiration of individual cells and bacteria, such as photosynthesis and gas exchange through the cell membrane^46,47^. Our sensor could also be applied to observe acoustic waves generated by the nanoscale vibrations associated with cellular metabolism^48^. Measurements of these vibrations have been shown to allow diagnostic assays of cellular toxicity and antibiotic resistance^48^, and provide insight into molecular processes such as conformational changes^49^. Unlike current atomic force microscope-based approaches^48^, our sensor could allow these measurements to be performed without physical contact, and therefore without disrupting the observed processes or contaminating the sensor. Moreover, the measurements could be performed with higher bandwidth, and resolve 100-picometre-level cellular vibration amplitudes at low kilohertz frequencies and sub-picometer vibrations at above 100 kHz (see Supplementary Note 9).

As with all ultrasonic sensors that use mechanical resonances (e.g.,^24–26^), one potential drawback of our sensor is that the best sensitivity is only achieved in narrow frequency windows near each mechanical resonance. This is not a concern for applications such as trace gas sensing and narrowband sonar where the signal is an acoustic tone of known frequency. In scenarios where broadband sensitivity is required, our approach has several attractive features compared to other resonant sensors. Firstly, the sensor is able to operate simultaneously on multiple mechanical resonances over the full 1 kHz to 1 MHz frequency band. Secondly, the combination of optical measurement and cavity enhancement provides a low shot noise floor, allowing high sensitivity even away from resonance. Finally, the cavity optomechanical architecture allows the use of techniques from quantum optomechanics to enhance the broadband response of future sensors^7^. For instance, the optical shot noise could be suppressed by engineering the cavity structure to increase the optomechanical coupling (such as in, e.g.,^40^) or using quantum correlated light^42^, laser cooling could be used to broaden and flatten the mechanical resonances without introducing additional thermal noise^13–16,50,51^ (see Supplementary Note 10), or laser levitated particles could be used to entirely remove substrate thermal noise^52^.

## Methods

### Device fabrication

The spoked microdisks were fabricated on silicon wafers, covered with a 1.8 μm layer of thermally grown silicon dioxide. The wafer was first coated with photoresist and spoked circular pads were defined using ultraviolet-photolithography (see Supplementary Fig. 10a). After developing the photoresist, the wafer was exposed to buffered Hydrofluoric acid, removing all the uncovered silicon dioxide (see Supplementary Fig. 10b). The remaining photoresist was consecutively cleaned off with acetone (see Supplementary Information Fig. 10c). In the subsequent step, the wafer was coated again with photoresist for protection and mechanically separated into about thirty chips containing ten circular silicon dioxide structures each. After separation, the photoresist was removed and the chips were individually exposed to XeF_2_ gas, selectively removing the silicon and releasing the silica structures (see Supplementary Fig. 10d, e).

### Characterisation setup

Light from a 1555 nm tunable Erbium-doped fibre laser [NKT Photonics, Koheras Adjustik] was guided to the experiment through an optical isolator to avoid reflection back into the laser. The intensity of the laser was adjusted using a variable fibre attenuator. The frequency of the laser could be thermally tuned over a range of about one nanometre or electronically swept over tens of picometers using a built-in piezo element of the laser cavity. The polarization of the light was adjusted using a fibre polarization controller. A tapered nanofibre was used to evanescently couple the laser to a whispering-gallery-mode of the disk. The crucial coupling distance between the taper and the disk was coarsely adjusted using manual micrometer stages and optical microscopes. Fine-tuning was implemented using a nanopositioning stage [Thorlabs MDT693A]. The transmitted light had intensity of around 20 μW and was detected with an InGaAs-photodetector [New Focus 1811 DC-125 MHz].

### Optical mode characterisation

The optical mode of the microdisk was investigated by measuring the transmission of the probe laser as a function of the laser frequency. The frequency of the laser (*λ* = 1555.716 nm) was swept over the cavity optical mode using a function generator and the probed laser transmssion was recorded with an oscilloscope (see Fig. 2c). The optical mode was found to have a quality factor *Q* = 1.8 × 10^6^ when the tapered fibre was positioned so that the input optical coupling rate matched the intracavity loss rate, i.e., critical coupling (see Supplementary Fig. 3). This implies an intrinsic quality factor of 3.6 × 10^6^.

### Noise floor and signal response

The high-frequency part of the signal was Fourier transformed in a spectrum analyser [Agilent N9010A] to analyse the sensor noise spectrum, and to calibrate the sensor SNR. The system network response was measured using a vector network analyser [Agilent E5061B] to determine the dependence of the SNR on applied acoustic pressure and to determine sensitivity as a function of frequency. The network analyser was also used to calibrate a piezo element (Thorlabs AE0505D08F) as a function of frequency and voltage to operate as the acoustic source (see Supplementary Note 5 for detail).

## Supplementary information

Supplementary Information

## Data Availability

The data that support the findings of this study are available within the paper and its Supplementary Information files.
