# Supplementary material for: Precision ultrasound sensing on a chip
Source: Nat Commun. 2019 Jan 10;10:132. doi: 10.1038/s41467-018-08038-4 (PMC6328601; doi:10.1038/s41467-018-08038-4)
Supplement: Supplementary file 1 — Supplementary Information [file 41467_2018_8038_MOESM1_ESM.pdf]

Supplementary information for:

Precision ultrasound sensing on a chip

Sahar Basiri-Esfahani, Ardalan Armin, Stefan Forstner, and Warwick P. Bowen

SUPPLEMENTARY NOTE 1. DERIVATION OF THE NOISE EQUIVALENT PRESSURE SENSITIVITY

We start by modelling the motion of a single mode mechanical oscillator at room temperature in response to an external acoustic drive, and probed by a coherent field. In regime where the mechanical thermal noise dominates on resonance, and the quantum back-action noise on the sensor is negligible, we can take the high temperature limit where $\bar{n}(\omega) = k_{\mathrm{B}}T/\hbar\omega$. In this regime, we obtain an optical shot-noise limited noise force floor. The Langevin equations of motion for the mechanical displacement and the optical cavity mode respectively are written as

$$m\frac{d^2x_{\mathrm{m}}(t)}{dt^2} + m\gamma\frac{dx_{\mathrm{m}}(t)}{dt} + kx_{\mathrm{m}}(t) = F_{\mathrm{T}} + F_{\mathrm{D}}(t), \tag{1}$$

$$\frac{da(t)}{dt} = -\frac{i}{\hbar}[a, H_{\mathrm{sys}}] - \frac{\kappa}{2}a(t) + \sqrt{\kappa_{\mathrm{in}}}a_{\mathrm{in}} + \sqrt{\kappa_{\mathrm{l}}}a_{\mathrm{l}}, \tag{2}$$

in which k is the spring constant, $F_{\mathrm{T}} = \sqrt{2m\gamma k_{\mathrm{B}}T}$ is the thermal force, γ is the mechanical damping rate, $F_{\mathrm{D}}(t) = r\zeta P_{\mathrm{D}}(t)A$ is the acoustic drive force in which r is the pressure participation ratio defined in the main text, P_D is the acoustic pressure, A is the sensing area, ζ quantifies the overlap of the displacement profile of the mechanical sensing element with the incident pressure wave, and m is the effective mass of the mechanical mode. κ_{in} is the input coupling of the cavity to the input optical field, κ_{l} is the intrinsic cavity loss and $\kappa = \kappa_{\mathrm{in}} + \kappa_{\mathrm{l}}$. Moreover, $H_{\mathrm{sys}} = \hbar\Delta_{\mathrm{c}}a^\dagger a + \hbar g_0 a^\dagger a x_{\mathrm{m}}$ is the Hamiltonian of the system in the interaction picture rotating with the frequency of the laser in which the optical detuning $\Delta_{\mathrm{c}} = \Delta + g_{\mathrm{disp}}x_{\mathrm{m}} + O^2 + ...$, includes the dispersive coupling due to the presence of the mechanical oscillation, and Δ is the optical detuning in absence of the mechanical oscillations. $\kappa_{\mathrm{in}} = \kappa_{\mathrm{in},0}(1 - g_{\mathrm{diss}}x_{\mathrm{m}})$ includes the dissipative coupling of the input field to the cavity in response to the acoustic field up to the first order in x_{m}. a_{in} and a_{l}, respectively, show the input optical field into the cavity and the vacuum input noise. In case where the input optical field is a semi-classical coherent laser field, we can displace the amplitude of the optical field such that $a \to \bar{a} + \alpha_{\mathrm{in}}$ where $|\alpha_{\mathrm{in}}|^2 = N$ is the input photon intensity.

The solution to Supplementary Equation (1) in Fourier transformed frequency domain is

$$x_{\mathrm{m}}(\omega) = \chi_{\mathrm{m}}(\omega)[F_{\mathrm{T}} + F_{\mathrm{D}}(\omega)], \tag{3}$$

in which the mechanical susceptibility χ_{m} is calculated as $\chi_{\mathrm{m}}^{-1} = m(\omega_{\mathrm{m}}^2 - \omega^2 - i\gamma_{\mathrm{m}}\omega)$, where m and ω_{m} are respectively the mass and the resonance frequencyof the mechanical

object. The output cavity mode, $a_{\rm out}$ is related to the cavity mode, a, and the input mode into the cavity, $a_{\rm in}$, through the input-output relation [13, 14], $a_{\rm out} = \sqrt{\kappa_{\rm in}}a - a_{\rm in}$. By solving Supplementary Equation (1), the motional displacement of the mechanical resonator can be calculated. Moreover, Supplementary Equation (2) can be solved in the frequency domain in linearised displacement regime to get the cavity mode a. Using the solutions to Supplementary Equations (1,2) together with the input-output relation, the output field of the cavity is calculated as

$$a_{\rm out}(\omega) = \left(B(\omega) - C(\omega)\right) x_{\rm m}(\omega) + D(\omega)a_{\rm in} + E(\omega)a_{\rm l}, \tag{4}$$

in which

$$\begin{aligned}
B(\omega) &= \frac{-2i\alpha_{\rm in}g_{\rm disp}\kappa_{\rm in,0}}{(\kappa_0 + 2i\Delta)(\kappa_0 + 2i(\Delta - \omega))}, \\
C(\omega) &= \frac{2\alpha_{\rm in}g_{\rm diss}\kappa_{\rm in,0}}{\kappa_0 + 2i(\Delta - \omega)}\left(1 - \frac{2\kappa_{\rm in,0}}{\kappa_0 + 2i\Delta}\right), \\
D(\omega) &= \frac{\kappa_{\rm in,0} - \kappa_l - 2i(\Delta - \omega)}{\kappa_0 + 2i(\Delta - \omega)}, \\
E(\omega) &= \frac{\sqrt{\kappa_{\rm in,0}\kappa_l}}{\kappa_0 + 2i(\Delta - \omega)},
\end{aligned} \tag{5}$$

in which $\kappa_{\rm in,0}$ is the original value of the input coupling in absence of the acoustic pressure, $\kappa_0 = \kappa_{\rm in,0} + \kappa_{\rm l}$, $g_{\rm disp} = \frac{d\Delta}{dx}$ is the dispersive coupling rate and $g_{\rm diss} = \frac{1}{\kappa_{\rm in,0}}\frac{d\kappa_{\rm in}}{dx}$ is the dissipative coupling rate. Hence, in the regime where $|\alpha_{\rm out}| \gg |\bar{a}|$, the output intensity of the cavity can be calculated as $I_{\rm out}(\omega) \sim \alpha_{\rm out}^* a_{\rm out}(\omega) + \alpha_{\rm out}a_{\rm out}^\dagger(-\omega)$ where $\alpha_{\rm out} = |\alpha_{\rm out}|e^{i\varphi}$ is the average amplitude of the output field. The intensity can be rewritten as $I_{\rm out}(\omega) \sim |\alpha_{\rm out}|X_{\rm out}^{\varphi}(\omega)$, in which $X_{\rm out}^{\varphi}(\omega)$ is defined as the amplitude quadrature of the output field fluctuations as $X_{\rm out}^{\varphi}(\omega) = a_{\rm out}(\omega)e^{-i\varphi} + a_{\rm out}^\dagger(-\omega)e^{i\varphi}$. As for the rest of the calculations we require the output fluctuations, we normalize the output intensity as $I_{\rm out}(\omega) \to \frac{I_{\rm out}(\omega)}{|\alpha_{\rm out}|} \sim X_{\rm out}^{\varphi}(\omega)$. For the case of having only dispersive coupling where we assume $g_{\rm diss} = 0$ we get

$$\begin{aligned}
X_{\rm out}^{\varphi}(\omega)|_{\rm disp} = {} & \chi_{\rm m}(\omega)(e^{-i\varphi}B(\omega) + e^{i\varphi}B^*(\omega))F_{\rm T} + \chi_{\rm m}(\omega)\zeta A(e^{-i\varphi}B(\omega) + e^{i\varphi}B^*(\omega))rP_{\rm D}(\omega) \\
& + |D(\omega)|X_{\rm in}^{\theta} + |E(\omega)|X_l^{\phi},
\end{aligned} \tag{6}$$

where we have used this convention in the Fourier frequency domain that $[a(\omega)]^\dagger = a^\dagger(-\omega)$. For the case of having only dissipative coupling where we assume $g_{\rm disp} = 0$ we have

$$\begin{aligned}
X_{\rm out}^{\varphi}(\omega)|_{\rm diss} = {} & \chi_{\rm m}(\omega)(e^{-i\varphi}C(\omega) + e^{i\varphi}C^*(\omega))F_{\rm T} + \chi_{\rm m}(\omega)\zeta A(e^{-i\varphi}C(\omega) + e^{i\varphi}C^*(\omega))rP_{\rm D}(\omega) \\
& + |D(\omega)|X_{\rm in}^{\theta} + |E(\omega)|X_l^{\phi},
\end{aligned} \tag{7}$$

where the area of the sensor, A, is the area of the disk.

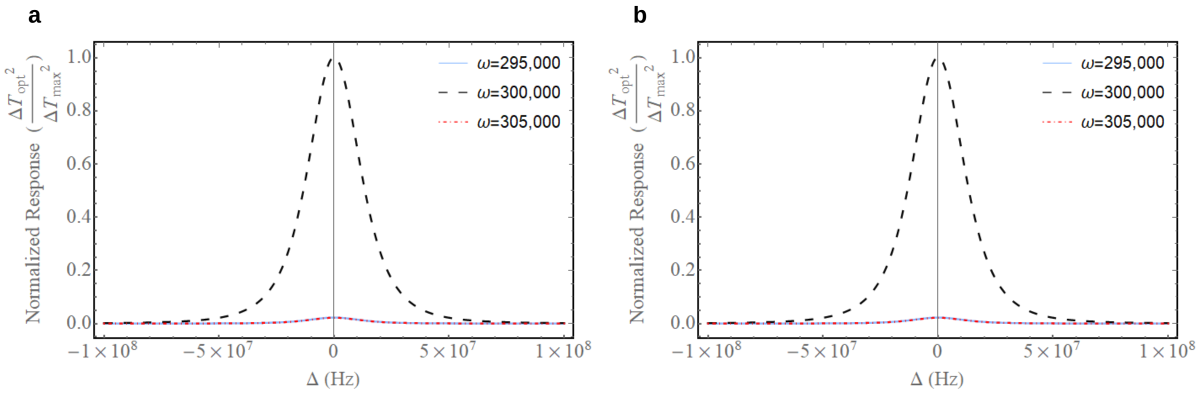

Supplementary Figure 1: Sensor response versus detuning of the laser form cavity resonance frequency. Plots are for $\kappa_{\mathrm{l}} = 4 \times 10^7$, $\kappa_{\mathrm{in}} = 0.5 \times 10^6$, and $\omega_{\mathrm{m}} = 300$ kHz at different frequencies (ω). (a) For the case where the sensor operates in pure dispersive optomechanical regime. (b) for sensor operating in pure dissipative coupling.

Based on the above mentioned convention, $[a(\omega)]^\dagger = a^\dagger(-\omega)$, the power spectrum of the observable, X_{out}, is defined as [15]

$$\begin{aligned} S_{X_{\mathrm{out}}X_{\mathrm{out}}}(\omega) &= \int_{-\infty}^{\infty} d\omega' \langle X_{\mathrm{out}}^\dagger(-\omega) X_{\mathrm{out}}(\omega')\rangle \\ &= \int_{-\infty}^{\infty} d\omega' \langle (a(\omega)a^\dagger(-\omega))(a^\dagger(-\omega')a(\omega'))\rangle. \end{aligned} \tag{8}$$

The power spectrum, $S_{X_{\mathrm{out}}X_{\mathrm{out}}}$, can be used to calculate the noise equivalent pressure sensitivity. Considering a signal to noise ratio (SNR) equal to unity, the noise power spectrum becomes

$$S_{\mathrm{PP}}[\mathrm{pa}^2\ \mathrm{Hz}^{-1}] = \frac{1}{r^2 A^2}(2m\gamma k_{\mathrm{B}}T + \frac{1}{N|\chi(\omega)|^2}), \tag{9}$$

where we define $\chi(\omega)$ as the optomechanical susceptibility, which depends on the particulars of the coupling regime. For dispersive coupling it is

$$\chi(\omega) = \frac{32 g_{\mathrm{disp}} \Delta \kappa_{\mathrm{in},0} \chi_{\mathrm{m}}(\omega)(\kappa_0 - i\omega)}{(4\Delta^2 + \kappa_0^2)(4\Delta^2 + (\kappa_0 - 2i\omega)^2)}, \tag{10}$$

and for dissipative regime it is

$$\chi(\omega) = \frac{\left[2 g_{\mathrm{diss}} \kappa_{\mathrm{in},0}(-\kappa_0(\kappa_{\mathrm{in},0} - \kappa_{\mathrm{l}})(\kappa_0 - 2i\omega) + 4\Delta^2(\kappa_0 + 2\kappa_{\mathrm{in},0} - 2i\omega)\right] \chi_{\mathrm{m}}(\omega)}{(4\Delta^2 + \kappa_0^2)(4\Delta^2 + (\kappa_0 - 2i\omega)^2)}. \tag{11}$$

For the valid regime in this work, $\omega \ll \kappa$, the optomechanical susceptibility reduces to a simpler form of

$$\chi(\omega) = \frac{2 g_{\mathrm{i}} \kappa_{\mathrm{in},0} \chi_{\mathrm{m}}(\omega)}{(4\Delta^2 + \kappa_0^2)^2} \times C^{\mathrm{i}}, \tag{12}$$

where $i \in \{\text{disp}, \text{diss}\}$, $C^{\text{disp}} = 16\kappa_0\Delta$ and $C^{\text{diss}} = -\kappa_0^2(\kappa_{\text{in},0} - \kappa_\text{l}) + 4\Delta^2(\kappa_0 + 2\kappa_{\text{in},0})$.

Sensor response

In Supplementary Equations (6) and (7), the coefficient in front of the external drive force by the applied pressure, $P_d(\omega)$, is the response of the system which is determined as the transmitted optical power through the system. In Supplementary Figure 1, we have plotted the system response versus detuning for both dispersive and dissipative regimes.

SUPPLEMENTARY NOTE 2. OVERLAP AND EFFECTIVE MASS FOR THE SECOND ORDER FLAPPING MODE

In order to compare the measured sensitivity near the second order flapping mode to theoretical predictions, it is necessary to estimate the overlap ζ between the applied acoustic pressure wave and the mechanical mode spatial profile, as well as the effective mass of the mechanical mode.

The overlap can be calculated as

$$\zeta = \int_A u(\mathbf{r})\delta p(\mathbf{r})dA, \tag{13}$$

where the integral is taken over the surface of the resonator; and $u(\mathbf{r})$ and $\delta p(\mathbf{r})$ are the mechanical displacement in the direction of the vertically incident pressure wave and the pressure acting on the resonator, respectively. $u(\mathbf{r})$ is normalized to equal unity at the maximum displacement of the mode and $\delta p(\mathbf{r})$ is normalized to the acoustic pressure at an antinode of the pressure wave.

The effective mass can be calculated as

$$m = t\rho \cdot \int_A |u(\mathbf{r})|dA, \tag{14}$$

where t is the thickness of the resonator, ρ is it's density, so that $M = tA\rho$ is the total mass of the resonator.

We used COMSOL multiphysics to determine $u(\mathbf{r})$ for the second order flapping mode. From Supplementary Equation (14) we then found that the effective mass of the mode was equal to about half the total mass of the resonator, $m \approx 0.5M \approx 110$ ng.

Considering that the pressure on the sensor, δp, is due to an incident plane wave, we found an overlap of approximately $\zeta = 0.14$ from Supplementary Equation (13).

One might expect this overlap to be zero, since the mode exhibits a rotational symmetric tilting motion around the axis of the device with downwards motion at the inner edge of the device at the same time as the outer edge is moving upwards. This counter-motion would lead to a cancellation of the force applied by a plane pressure wave. However, the node of the mode is closer to the outer edge than the center-of-mass of the annulus, leading to a residual center-of-mass motion and a non-vanishing overlap.

SUPPLEMENTARY NOTE 3. FLUIDIC DAMPING OF MICROMECHANICAL DEVICE

In this section we outline expressions and describe an experiment that allows the fluidic damping due to the interaction of our spoked-disk micromechanical resonator with its gaseous environment to be estimated. We follow reference [17]. There are three common forms of fluidic damping relevant to micromechanical devices: ballistic damping, due to collisions of gas molecules with the surface of the resonator; drag force damping, due to viscousness of the gas and the velocity gradient between the boundary layer near the surface of the resonator and more distant points in the fluid; and squeeze-film damping, due to the change in pressure introduced by motion of the resonator near its substrate. Ballistic damping is generally only significant in high vacuum conditions, and is therefore not considered further here. The other two forms of damping, in general, introduce a combined force that opposes the velocity of the resonator. Following Chapter 3 in reference [17], and treating the resonator as thin, so that the spatiotemporal eigenmodes $\mathbf{u}(x, y, z, t)$ which describe the displacement of each small region of the resonator vary only in the plane of the resonator (defined as the $\{x, y\}$ plane here), and do not depend on the z-coordinate (i.e. $\mathbf{u}(x, y, z, t) = \mathbf{u}(x, y, t)$), this force can be written as $F = -\mu l \dot{\mathbf{u}}(x, y, t)$ where μ is the coefficient of viscosity of the fluid and l is a geometry-dependent characteristic length-scale to be determined later. To find the rate of damping due to the gas γ_{gas}, this force should be compared to the acceleration of the resonator $F_{\text{accel}} = m\ddot{\mathbf{u}}(x, y, t)$, where m is the effective mass of the mode described by $\mathbf{u}$. Considering the acceleration and damping terms in the general equation of motion for harmonic oscillation $m\ddot{x} + m\gamma\dot{x} + kx = F_{\text{ext}}$, where k is the

spring constant and F_{ext} the external force, we see that γ_{gas} is given simply by

$$\gamma_{\text{gas}} = \mu l/m, \tag{15}$$

in angular units.

The power spectral density of the thermal force noise introduced by fluctuation-dissipation to complement this fluidic dissipation is

$$S_{T,\text{gas}} = 2m\gamma_{\text{gas}} k_B T = 2\mu l k_B T, \tag{16}$$

where, of course, k_B is the Boltzmann constant, and T is the temperature of the system. Since the incident acoustic wave travels within the gaseous medium, $S_{T,\text{gas}}$ presents a fundamental bound on the pressure sensitivity of a micromechanical acoustic sensor of fixed geometry and a particular gaseous medium.

Given that the thermal fluctuations introduced by the interaction with the gas are independent from those introduced by thermal vibrations of the substrate and any other damping mechanisms intrinsic to the resonator, the total thermal force noise experienced by the resonator is

$$S_T = 2m(\gamma + \gamma_{\text{gas}})k_B T = 2(m\gamma + \mu l)k_B T, \tag{17}$$

where γ is the intrinsic decay rate of the resonator, and the total mechanical decay rate $\gamma_m = \gamma + \gamma_{\text{gas}}$. Inserting this expression into Supplementary Equation (9) we find the minimum detectable pressure

$$P_{\min}(\omega) = \sqrt{S_{\text{PP}}(\omega)} = \frac{1}{r\zeta A}\sqrt{2(m\gamma + \mu l)k_B T + N^{-1}|\chi(\omega)|^{-2}}, \tag{18}$$

as given in the main text, where r is the pressure participation ratio (see main text Figure 2d), and ω is the drive frequency of the acoustic wave. Note that, since the sensor and detection system are linear, the effect on inefficiencies in detection are simply to transform the effective intracavity photon number from $N \to \eta N$ where η is the efficiency with which light leaves the optical resonator and is successfully detected at the detector.

Experimental characterisation of the gas damping

In order to determine the contributions to the noise equivalent pressure from intrinsic mechanical dissipation and from fundamental gas damping, we placed the device in a vacuum

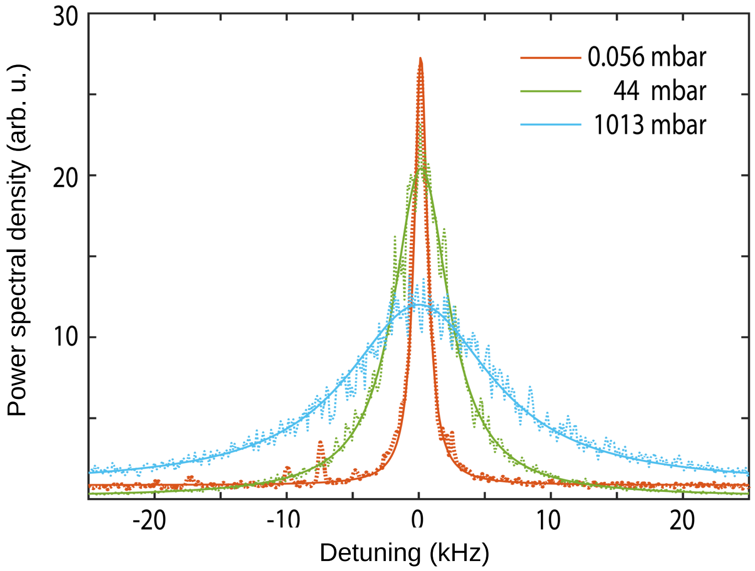

Supplementary Figure 2: Brownian noise spectra of a mechanical resonance with varying static pressure. Power spectral density versus detuning around the 315 kHz resonance for different pressures with decay rates of 1430 Hz, 535 Hz and 150 Hz at pressures of 1000 mbar, 44 mbar and 0.056 mbar respectively. Note that the vertical axis of this plot is uncalibrated, and varied for measurements at different pressures due to drifts in the experimental apparatus, including the taper-microdisk separation and the optical polarisation.

chamber and swept the pressure from 0.056 mbar to atmosphere. We monitored the damping rate of the resonance observed at 315 kHz. At the lowest measured pressure, the decay rate plateaus to a minimum of 150 Hz, corresponding to the intrinsic mechanical dissipation γ, whereas at atmospheric pressure, the decay rate reaches $1,430$ Hz. The difference between these two values corresponds to a gas damping rate of $\gamma_{\rm gas}/2\pi = 1,260$ Hz. Mechanical resonances for three different pressures, measured using a spectrum analyser, are shown in Supplementary Figure 2.

The contributions to the noise equivalent pressure from intrinsic dissipation and gas damping could potentially also be distinguished by varying the viscosity of the gas in other ways; for instance by changing the constituents or temperature of the gas. Evacuating the sample chamber is particular attractive because it suppresses the gas damping by several orders of magnitude without affecting the intrinsic dissipation, and therefore allows a direct and accurate measurement of the intrinsic dissipation. By contrast, decreasing the temperature of the enclosure by 100 degrees to ∼200 K would only reduce the air viscosity by

a factor of two. Similarly, replacing the air with alternative gas such as carbon dioxide, hydrogen, helium or xenon would also only alter the viscosity by a factor of two or less. A further complication associated with changing the temperature is that the intrinsic mechanical dissipation is also temperature dependent, for instance decreasing by roughly a factor of four with a 100 degree decrease in temperature for devices similar to ours in Ref. [19].

Determining the characteristic length-scale

From the experimentally observed gas damping of $\gamma_{\text{gas}}/2\pi = 1,260$ Hz we can directly determine a viscous length-scale $l = 2\pi \cdot 8.1$ mm. The length scale has two components – one from drag damping (l_{drag}), and one from squeeze film damping (l_{squeeze}). While the viscous drag damping can be reliably calculated for our geometry, as shown in the following paragraphs, the viscous squeeze film damping depends sensitively on the height profile of the underlying substrate. In our case, large height variations arise in the silicon substrate below the sensor due to our fabrication process, as can be seen in Figure 2a of the main text. This makes the exact determination of the viscous squeeze film damping extremely challenging, and beyond the scope of this work. We instead infer the characteristic length scale for squeeze film damping from the experimentally extracted total gas damping and the calculated viscous damping.

Determining the viscous drag length scale

Chapter 3 of Ref. [17] gives the drag force for several geometries, with the general form

$$F_{\text{drag}} = -6\pi\xi\mu\sqrt{A}\,\dot{\mathbf{u}}(x, y, t), \tag{19}$$

where, again, μ is the coefficient of viscosity of the fluid, which for air at room temperature is around $\mu = 1.8\times10^{-5}$ kg m^{-1} s^{-1}, A is the surface area of the top surface of the resonator and ξ is a dimensionless geometry dependent coefficient which is generally on the order of unity. For instance, for a free sphere, a vertically moving disk (z direction), and a horizontally moving disk, it is given by $\xi = \{1, 0.85, 0.567\}$, respectively.

From Supplementary Equation (19), we find

$$l_{\mathrm{drag}} = 6\pi\xi\sqrt{A} = 6\pi^{3/2}\xi\sqrt{R^2 - r^2} \approx 3\pi\left(2\xi\sqrt{A}\right), \tag{20}$$

$$\gamma_{\mathrm{drag}} = \frac{\mu l}{m} = \frac{3\pi\mu}{m}\left(2\xi\sqrt{A}\right). \tag{21}$$

It should be noted that in these expressions m is the effective mass of the mechanical eigenmode, which we determine to be $m \sim 110$ ng $\sim M/2$ from finite element modelling, while the drag damping calculations assume a uniform vertical motion of the disk - that is, it does not account for any structure in the mechanical modeshape.

Calculations that fully account for the modeshape dependence of gas damping are beyond the scope of this work. However, it can be approximately accounted for via a simple thought experiment. We imagine that the component of the annular disk is such that the surface of the disk within a radius r' of its axis is perfectly stationary, while the component between r' and the major radius of the disk R moves uniformly. That is, the mechanical eigenmode is a step-function in the radial direction, with no motion at radii less than r'. In this case, the effective mass of the resonator is equal to

$$m = M \times \frac{A'}{A} = M \times \left(\frac{R^2 - r'^2}{R^2 - r^2}\right), \tag{22}$$

where A' is the area of the moving component of the annular disk and M is the total mass of the annular disk.

Since the moving component of the annular disk is also an annular disk with the same major radius R, but minor radius increased to r', Eqs. (20) and (21) can be applied to approximate the drag damping and its characteristic length scale, but with the replacement $A \to A'$. We then arrive finally at

$$l = l_{\mathrm{drag}} \approx 6\pi\xi\sqrt{\frac{Am}{M}}, \tag{23}$$

$$\gamma_{\mathrm{drag}} = \frac{\mu l_{\mathrm{drag}}}{m} \approx \frac{6\pi\mu}{m}\xi\sqrt{\frac{Am}{M}}. \tag{24}$$

The relevant parameters of our device are $\mu = 1.8 \times 10^{-5}$ kg m^{-1} s^{-1}, $R = 148$ µm, $r = 82$ µm, a mass of around $M \sim \rho t A = 230$ ng, and an effective mass of $m \sim M/2$, where $\rho = 2650$ kg m^{-3} is the density of silica, $t = 1.8$µm the thickness of the resonator, and A its area. We choose $\xi = 0.85$, consistent with expectations for a vertically moving disk.

In order to state a sensitivity in units of Pa Hz$^{-1/2}$, where Hz is the inverse of the actual measurement time, we include an extra scaling factor of 2π to convert from radial units that are used in the derivations above: $l \to l/2\pi = m\gamma/2\pi\mu$. We then find

$$l_{\text{drag}} \sim 0.4 \text{ mm}, \tag{25}$$

$$\gamma_{\text{drag}}/2\pi \sim 62 \text{ Hz}. \tag{26}$$

From this, we infer an approximate squeeze film damping characteristic length scale of $l_{\text{squeeze}} = l - l_{\text{drag}} = 7.7$ mm $\gg l_{\text{drag}}$, suggesting that squeeze film damping is dominant, and thus the total gas damping could be significantly reduced by increasing the distance between the sensor and the substrate.

Squeeze film damping in the presence of a flat substrate

In this Section we will derive an approximate expression for the squeeze film damping above a flat substrate. While, due to the large height variations of the substrate across the area of our device, this analysis can not be used to determine l_{squeeze}, we can use it to assess how an optimized sensor geometry could be designed.

Ref. [17] calculates the squeeze film force for a vertically moving annular disk with major and minor radii of R and r, respectively, to be

$$F_{\text{squeeze}} = -\frac{3\pi\mu R^4 G(\beta)}{2h^3}\,\dot{\mathbf{u}}(x, y, t), \tag{27}$$

where h is the separation of the bottom of the resonator from the substrate, $\beta = r/R$ and

$$G(\beta) = 1 - \beta^4 + \frac{(1-\beta^2)^2}{\ln\beta}. \tag{28}$$

Using these expressions, we find directly that

$$l_{\text{squeeze}} = \frac{3\pi R^4 G(\beta)}{2h^3}. \tag{29}$$

To account, roughly, for the eigenmode shape of the mechanical mode of the sensor, following the approach taken in the previous section, we can redefine a modified β as

$$\beta' = \frac{r'}{R} = \sqrt{1 - \frac{A}{\pi R^2}\frac{m}{M}} = \sqrt{1 - (1-\beta^2)\,\frac{m}{M}}, \tag{30}$$

where we have used the fact that $A/\pi R^2 = 1 - \beta^2$. We then find

$$l_{\text{squeeze}} \approx \frac{3\pi R^4 G(\beta')}{2h^3}, \tag{31}$$

$$\gamma_{\text{squeeze}} \approx \frac{3\pi \mu R^4 G(\beta')}{2mh^3}. \tag{32}$$

The relations derived in this Section for the characteristic length scales and damping rates for air-drag damping and squeeze-film damping, combined with modelling of the intrinsic mechanical damping, such as that performed in Ref. [18], allow approximate prediction of the thermomechanical noise limited sensitivity of a general spoked-microdisk cavity optomechanical acoustic sensor.

Designing the device to reach optimum sensitivity

As given in Supplementary Equation (16), the thermal force noise introduced by fluidic dissipation, and therefore the gas-damping limited pressure sensitivity, only depends on the Boltzmann constant, the temperature, the gas viscosity and the characteristic length-scale. Therefore, the characteristic length-scale is the only device-dependent parameter which can be engineered to optimise the sensitivity in this limit. The right-hand-side of Supplementary Equation (23) represents the characteristic length-scale for air-drag damping. As can be seen, apart from the geometric-factor ξ this depends only on the area of the resonator which is fixed for a given desired spatial resolution and the ratio of mass to effective mass. The air-drag damping limited sensitivity is therefore also fixed for given desired spatial resolution and device geometry. On the other hand, the squeeze-film damping characteristic length-scale (right hand side of Supplementary Equation (31)) depends on the height of the device above the substrate h, decreasing with increasing height. The two characteristic length-scales are equal for $h = (R^4 G(\beta')/(4\xi\sqrt{Am/M}))^{1/3}$. Taking the rough approximation that $A \sim R^2$, $m = M$, and $\beta \sim 0$ so that the hole in the annular disk is small relative to its diameter, this becomes $h \sim R$. We therefore see that squeeze-film damping can be expected to significantly degrade the pressure sensitivity if the height of the device is small compared to its radius. This is the case for our existing devices as can be seen from Figure 2a in the main text, consistent with our observation above that squeeze film damping dominates drag damping for our devices. Given the $1/h^3$ scaling in Supplementary Equation (31), it is clear that by developing a modified fabrication process that allow our devices to be suspended

further from the substrate, the squeeze-film damping could be greatly suppressed.

It is interesting to also observe that l, and therefore the thermal force noise from gas damping, is independent of the thickness of the device. On the other hand, as can be seen in Supplementary Equation (17), the force noise due to intrinsic damping into the substrate increases linearly with thickness, through the increase this causes to the device mass. Consequently, as the resonator becomes increasingly thin, and the intrinsic thermal force noise decreases, the noise introduced by gas-damping will become dominate (as is already the case for our devices). For sufficiently good optical measurement and a sufficient height above the substrate, this would allow the sensor to operate at the air-drag damping force noise floor. Indeed, for a sufficiently thin device, it may be possible to achieve an air-drag damping-limited noise floor even without the presence of spokes to isolate the device from substrate thermal noise. In this case, the active sensing would be increased by around 40% improving the sensitivity by a similar margin.

SUPPLEMENTARY NOTE 4. CHARACTERISING THE OPTICAL RESONANCE

The optical resonance used in the experiments was characterised by scanning the frequency of the laser across the mode and fitting the observed transmission through the tapered fibre to an inverted Lorentzian (see Supplementary Figure 3). This allowed the coupled cavity decay rate κ and quality factor Q to be determined. These were found to be $\kappa = 112$ MHz and $Q = 1.8 \times 10^6$ in critical coupling regime corresponding to an intrinsic quality factor of 3.6×10^6.

SUPPLEMENTARY NOTE 5. UTILISING A MICHELSON INTERFEROMETER TO CALIBRATE THE PIEZOELECTRIC SOUND SOURCE

To calibrate the piezo element (PZT1), we attached to it a light weight silver mirror (M_1) which is utilized as a mirror to be displaced in one of the arms of a Michelson interferometer as shown in Supplementary Figure 4a. The interferometer is fed by a laser at $\lambda \simeq 1555$ nm and the output interference signal is detected on a low noise photodetector as shown in the experiment scheme. We used a secondary PZT element (shown as Phase control PZT in

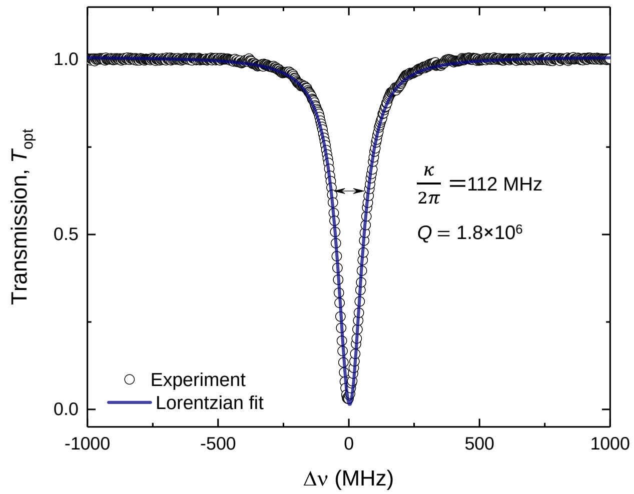

Supplementary Figure 3: Optical resonance. One of the optical modes of the microdisk ($\lambda = 1555.716$ nm) is shown with optical quality factor (Q) and cavity damping rate (κ) indicated on the figure. $\Delta\nu$ is the laser detuning with respect to the center of the cavity mode.

Supplementary Figure 4a) with a mirror attached to it in the other interferometer arm to thermally lock its phase using a PID (proportional integral derivative) controller.

To perform the measurement, we used a network analyser with its port 1 driving the PZT1 (DUT) and port 2 receives the signal from the PD. The PI-controller output is connected to PZT2 (phase control) through a voltage amplifier (Falco Sytems WMA-300). The displacement spectrum of PZT1 can be calculated as

$$d(\omega) = \frac{\lambda}{4}\frac{V(\omega_{\mathrm{ref}})}{V_{\mathrm{max}}}\sqrt{\frac{S_{21}(\omega)}{S_{21}(\omega_{\mathrm{ref}})}}, \tag{33}$$

where $S_{21}(\omega)$ is the off-diagonal network scattering parameter corresponding to the coherent power transfer from port 1 to port 2 at a frequency ω. ω_{ref} is a calibration reference frequency which was 20 kHz in our measurement. $V(\omega_{\mathrm{ref}})$ is the photodetector voltage at ω_{ref} and V_{max} is the maximum voltage generated by the interference, corresponding to a $\frac{\lambda}{4}$ displacement. Throughout this measurement we always monitored the generated signal not to saturate i.e., the displacement was always $< \frac{\lambda}{4}$ at a given applied voltage to the PZT1. We confirmed that the displacement was a linear function of the applied voltage to PZT1. At frequencies where the displacement was larger than $\frac{\lambda}{4}$, we lowered the voltage in order

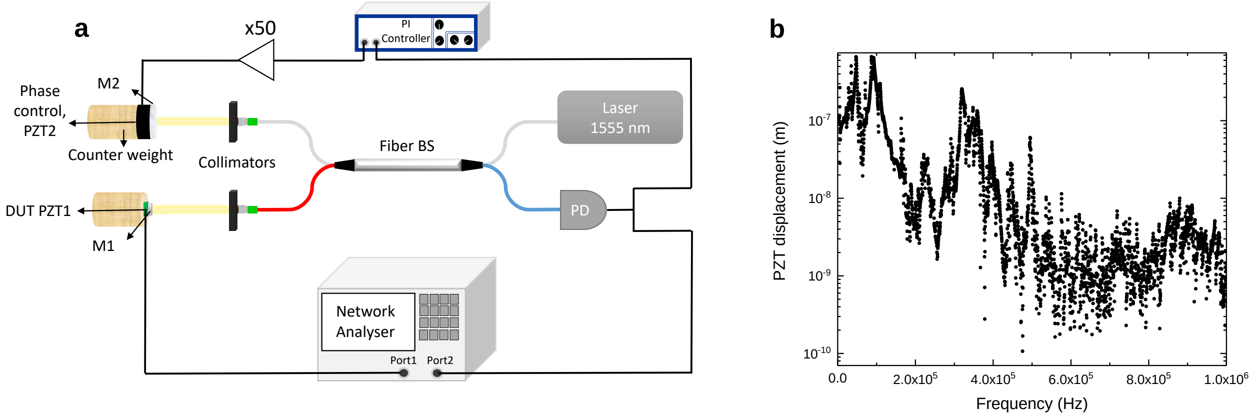

Supplementary Figure 4: Michelson interferometer to calibrate the piezo-electric sound source. (a) Interferometry measurement scheme. There are two mirrors M_1 and M_2 which are respectively attached to PZT1 and PZT2 which are placed in the output arms of the interferometer. PZT1 is the piezo element to be measured and PZT2 is used to lock the phase of the interferometer. PZT1 and PD are respectively connected to ports 1 and 2 of a NA with which we drive the PZ1 and simultaneously measure the signal of the interferometer. (b) The measured displacement of the PZT1 for a drive voltage of 707 mV at frequency spectrum from 1 kHz to 1MHz.

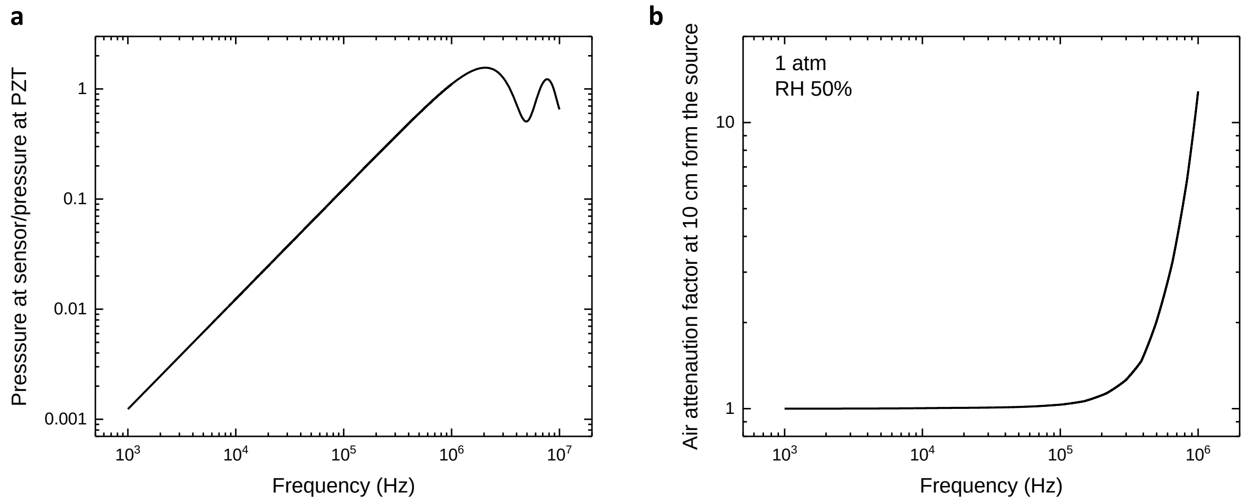

Supplementary Figure 5: Air acoustic response. (a) atmospheric attenaution for sensor-PZT distance of 10 cm based on citation [16], (b) the ratio of pressure at the position of the sensor to ultrasonic pressure at the PZT calculated from on-axis diffraction for sensor-PZT distance of 10 cm.

to avoid saturation. The high and low voltage measurements were then normalised with respect to 707 mV and compiled. The results are shown in Supplementary Figure 4b for an applied voltage of 707 mV.

The acoustic pressure at the position of the PZT is calculated from the PZT displacement and the air impedance, α=413 Pa s m^{-1} as

$$P_{PZT}(\nu) = \pi\nu d(\nu)\alpha, \tag{34}$$

and the acoustic pressure at the position of the sensor is given by

$$P_{sensor}(\nu) = c(\nu)\gamma(\nu)^{-1}P_{PZT}(\nu), \tag{35}$$

where c is an attenuation coefficient dependent on the sensor-PZT distance (L). We calcaulted this factor based upon on-axis diffraction of a plane wave from the PZT mirror which acts as an apperture through which the sound wave is diffracted. In our measurement the PZT-sensor distance was 10 cm and the appreture size was 7 mm x 7 mm. The results of this calculation as a function of ultrasonic frequency is shown in Supplementary Figure 5a. $\gamma(\nu)$ is the atmospheric acoustic attenuation and depends on both L and air acoustic absoprtion coefficient [16] which is significant only at high frequencies (>100kHz) for small L=10 cm. This attenuation factor is shown in Supplementary Figure 5b.

SUPPLEMENTARY NOTE 6. SENSITIVITY AND RESPONSIVITY

The experimentally measured pressure sensitivity (black line and blue circles) and the applied pressure (red line) are shown as a function of frequency in Supplementary Figure 6. The sensitivity is calculated using Equation (2) in the main text and using the measured responsivity and noise floor of the device. The experimentally measured responsivity of the sensor in VPa^{-1} is shown in Supplementary Figure 7. As can be observed and might be expected, the response is stronger at low frequencies though, as can be seen in the main text, the noise floor is also increased at low frequencies due to $1/f$ noise. The response also exhibits sharp resonances, as expected for a resonantly enhanced sensor.

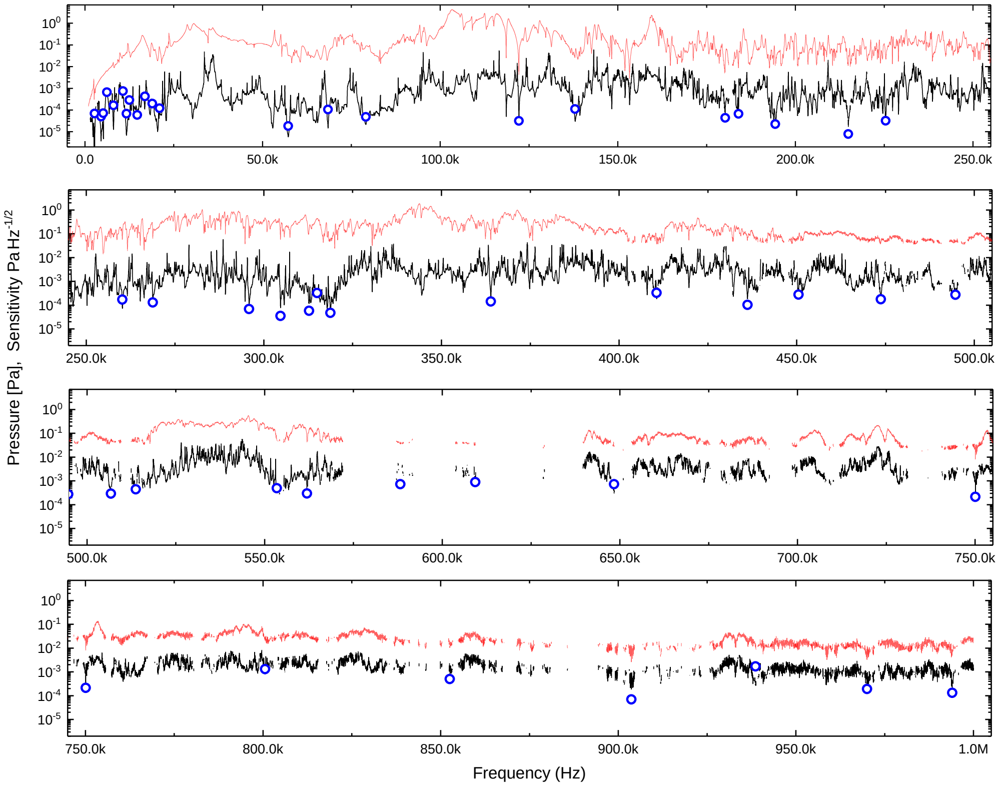

Supplementary Figure 6: Noise equivalent pressure sensitivity. Pressure sensitivity as a function of frequency (black lines) and the applied pressure (red lines) as measured using a network analyser. The open symbols are reference points directly measured using a spectrum analyser at certain frequencies for further validation of the network analysis.

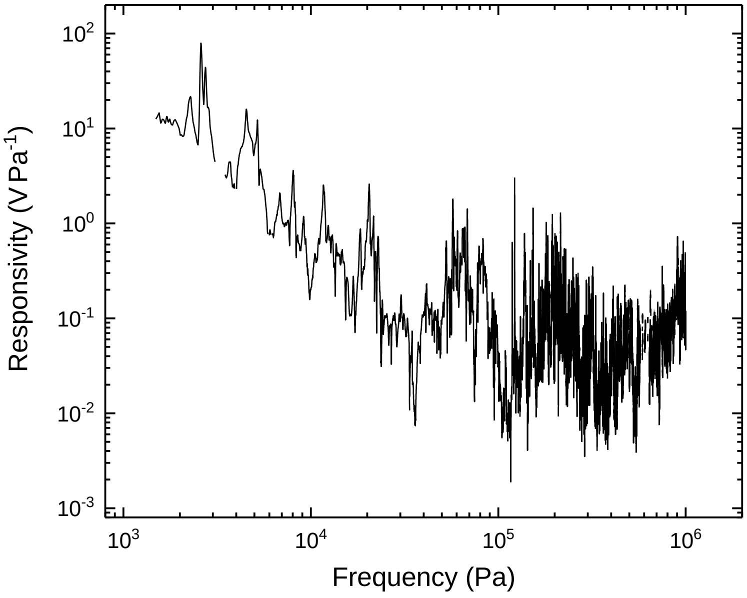

Supplementary Figure 7: Acoustic response. Responsivity of the sensor in V Pa^{-1}.

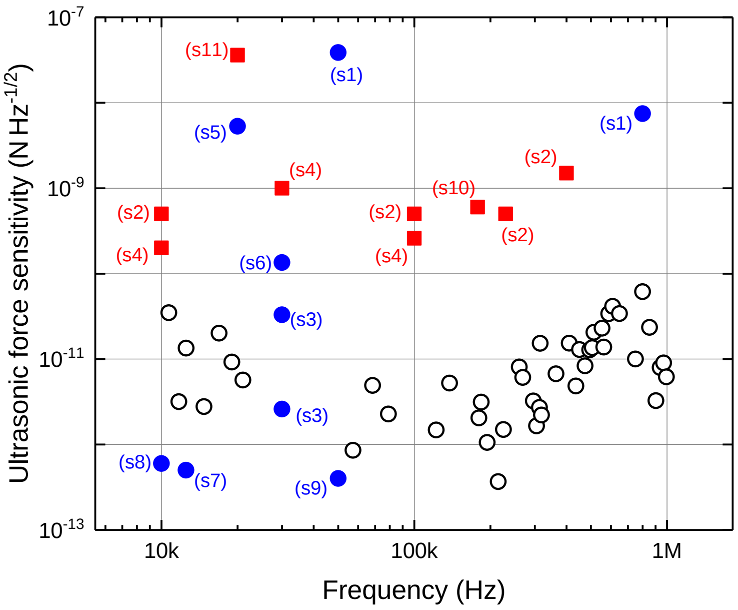

Supplementary Figure 8: Ultrasonic force sensitivity in comparison with other air-coupled sensors. s1 to s11 refer to Supplementary references [1]–[11].

SUPPLEMENTARY NOTE 7. PREVIOUS ULTRASOUND SENSORS

References for Figure 5 in the main text

Supplementary Figure 8 provides citations to previous works on acoustic sensors.

Effective area and ultrasonic force sensitivity of Fabry-Perot style acoustic sensor

Apart from the Fabry-Perot style acoustic sensor of [12], the area used to normalise the ultrasonic force sensitivity of the sensors discussed in the main text can generally be unambiguously defined. In the case of [12], however, the different sensing mechanism, optical detection of the refractive index modulation the pressure wave induces in a gas, makes the appropriate definition less clear. To clarify this, here we consider the diffraction of an acoustic wave incident on their sensing head.

The sensor head in [12] consists of a semi-enclosured space, with two vertical surfaces serving as mirrors to define the Fabry Perot cavity and two horizontal surfaces consisting of spacers to support the cavity. The final two faces of the enclosure are left open. The

system is then immersed in liquid, with an applied plane acoustic wave propagating into the sensing region (the locality of the optical field within the cavity) through the open faces. The acoustic wave modifies the refractive index of the enclosed gas and therefore the optical path length in the cavity. The total field of view for the acoustic wave is approximately 2 mm by 2 mm. The wave propagates for roughly 2 mm within the enclosure before reaching the axis of the laser beam.

We would like to know how the amplitude of the acoustic wave reaching the axis of the laser beam, and therefore the signal-to-noise, would change due to diffraction if, rather than a plane acoustic wave covering the full field of view of the sensor, the acoustic wave was concentrated on a smaller area of the outer surface of the spacer. If that area matches the cross sectional area of the laser beam through which the acoustic wave propagates (2 mm by 60 μm) and it is found that the amplitude is approximately unchanged by diffraction, the laser beam cross section would be the appropriate sensing area to choose. On the other hand, if diffraction significantly decreases the amplitude of the acoustic wave when it is concentrated to an area matching the laser beam cross section, the appropriate sensing area can be found by increasing the concentration area until there is no significant diffraction. Put another way, were the plane incident wave reduced in area, then for areas for which diffraction is small, this would leave the pressure at the sensing region, and therefore sensitivity, roughly unchanged. However, once the the incident plane wave area is reduced to the point where diffraction plays a significant role, the pressure at the sensing region would decrease for a fixed incident intensity, degrading the sensitivity.

To estimate the diffraction within the spacer we consider diffraction of a wave with Gaussian profile, noting that this gives a minimum possible diffraction (e.g. the perhaps usual square-profile would diffract faster). The diffraction length is then quantified by the Rayleigh length

$$z_R = \frac{\pi w^2}{\lambda}, \tag{36}$$

where w is the radius of the acoustic wave incident on the outside of the spacer and λ its wavelength. Assuming that the liquid in which the sensor is immersed is water, the longitudinal sound velocity is $v = 1,500$ m s^{-1}. For their 1 MHz acoustic wave frequency, this gives a wavelength of $\lambda = v/f = 1.5$ mm. We then ask, what radius of acoustic wave would be required for it to not diffract significantly over the 2 mm propagation distance to

the laser beam axis? This is given by setting the Rayleigh length equal to 2 mm, so that

$$w = \sqrt{\frac{z_R \lambda}{\pi}} \sim 1 \text{ mm}, \tag{37}$$

or an acoustic wave diameter of 2 mm. This implies that even an acoustic wave fully spanning the 2 mm by 2 mm field of view of the sensor would experience significant diffraction propagating through the spacer, and indeed given that the incident pressure wave profile will not be Gaussian, that the effective area of the sensor is likely to be larger than the field of view. To be conservative, in Figure 5 of the main text we choose the effective area to match the field of view.

Given the reported noise equivalent pressure of 0.45 mPa $\text{Hz}^{-1/2}$ in Ref. [12], we then arrive at an ultrasonic force sensitivity of 1.8 nN $\text{Hz}^{-1/2}$.

SUPPLEMENTARY NOTE 8. ESTIMATION OF SENSITIVITY OF TRACE GAS SENSING BY PHOTO-ACOUSTIC SPECTROSCOPY

Photo-acoustic gas spectroscopy is based upon sensing the acoustic waves generated by gas moleucles due the light absoprtion. Excitation light is properly chosen to be on resonance with one of the spectral lines of the gas molecules. Absorption of light in the gas produces local heating in the sample which results in local pressure increase. If the excitation light is pulsed or a modulated continuous-wave (CW), the generated heat in the gas will result in generation of acoustic waves at the modulation frequency. Photo-acoustic gas sensing is based on measuring the generated acoustic pressure to measure the gas absorption and so the concentration of the sample gas. The optomechanical sensor has high sensitivity together with microscale area. Hence, it offers the possibility to image gas concentrations with high resolution. Here we consider one example, the possibility to measure the CO_2 exchange of photosynthetic cells.

We can estimate the lowest gas concentration which can be measured by the optomechanical acoustic sensor in vicinity of a photosynthetic sample such as a plant leaf [20]. For this. we need to connect the minimum detectable pressure by the opto-mechanical microphone to the optical absorption coefficient in order to calculate the minimum of gas concentration which can be measured. We consider a microscale photosynthetic sample which exchanges CO_2 with its environment. We place the acoustic sensor at a distance r from the sample

and shine a pulsed laser through the gas in the vicinity of the sample. We choose the spectral line of CO_2 at $\lambda = 4,329.93$ nm ($k = 2311.105$ cm^{-1}) having line intensity of $S = 4.7 \times 10^{-19}$ cm^{-1}(molec cm^{-2})$^{-1}$.

For the remainder of the analysis we follow [21] to find the relation between absorption coefficient of the gas and the generated photoacoutic pressure. As in [21], we consider a thin optical medium (low absorption) for which $\alpha l \ll 1$ where α is the optical absorption coefficient and l is irradiation length or length of the photoacoustic source. We further assume that the sound wave can exit the irradiated zone within the pulse duration so that $R_{\mathrm{s}} < v\tau_{\mathrm{L}}$, where R_{s} is the radius of the laser beam, v is the speed of sound and τ_{L} is the laser pulse duration. Therefore, the effective source radius is $R = v\tau_{\mathrm{L}}$. The second assumption can therefore be rewritten as $R_{\mathrm{s}} < R$, i.e. that the radius of the laser beam should be smaller than the source radius. Moreover, the source volume can be written as $V = \pi R^2 l$. The coefficient of expansion of air, β is

$$\beta = \frac{\Delta V}{V \Delta T}, \tag{38}$$

where $\Delta V = \pi (R + \Delta R)^2 l$ is the initial expansion of the source volume after the laser beam, and ΔT, the rise in the temperature after a pulse, is [21]

$$\Delta T = \frac{E \alpha l}{\rho V C_p}, \tag{39}$$

in which E is the energy of the laser pulse, ρ is the density and C_p is the heat capacity of air. Therefore, ΔR, the initial expansion of the source radius becomes [21]

$$\Delta R = \frac{\beta E \alpha}{2\pi \rho C_p v \tau_{\mathrm{L}}}. \tag{40}$$

The peak displacement, $U_s(r)$ at distance r from the photoacoustic source varies as $\frac{1}{r}$ for spherical sound waves. Hence,

$$U_s(r) = \Delta R(\frac{R}{r}) = \frac{\beta E \alpha}{2\pi \rho C_p r}. \tag{41}$$

the peak acoustic pressure is [21]

$$P_{\mathrm{peak}}(r) \approx \frac{v \rho U_s(r)}{\tau_{\mathrm{L}}}. \tag{42}$$

Supplementary Equations (41) and (42) result in

$$\alpha \approx \frac{2\pi C_p \tau_{\mathrm{L}} r}{v \beta E} P_{\mathrm{peak}}(r). \tag{43}$$

To calculate the minimum detectable concentration we first need to relate the peak acoustic pressure to the effective acoustic pressure driving the mechanical mode over a period of the mechanical motion. The conversion factor can be estimated as the ratio of the laser pulse duration and the mechanical period over which the pressure is being applied. We have

$$P_{\text{eff}}(r) = P_{\text{peak}}(r)\tau_{\text{L}}\frac{\omega_{\text{m}}}{2\pi}, \tag{44}$$

in which ω_{m} is the mechanical frequency. Moreover, the absorption coefficient is proportional to gas concentration as

$$\alpha = \frac{cS}{2\gamma_G}, \tag{45}$$

in which $S = 4.7 \times 10^{-19}$ cm^{-1}(molec cm^{-2})$^{-1}$ is line intensiy of CO_2, $\gamma_G = 0.06$ cm^{-1} is gas linewidth of and c is the number density of gas molecules. Using Supplementary Equations (43), (44) and (45) we can write

$$c_{\text{min}} \approx \frac{8\pi^2\gamma_G C_p r}{v\beta E S\omega_{\text{m}}} P_{\text{eff}-\text{min}}, \tag{46}$$

where $P_{\text{eff}-\text{min}}$ is the minimum pressure that can be detected by the optomechanical transducer and c_{min} is the minimum detectable gas molecules number density. At room temperature, $T = 300$ K, $\beta = 0.0034$ K^{-1} and $C_p = 1.005$ kJ kg^{-1} K^{-1}. We further assume a pulsed laser having a pulse energy of $E = 1$ μJ and a pulse duration of $\tau_{\text{L}} = 1$ μs. This pulse duration is short enough to satisfy the condition of $R_{\text{s}} < v\tau_{\text{L}}$ for a typical laser beam radius of $R_{\text{s}} = 50$ μm but also long enough to avoid thermal diffusion during the exposure. If we choose the acoustic frequency of $\nu_0 = 318$ kHz at which the optomechanical sensor can detect acoustic pressures as small as $P_{\text{min}} = 84$ μPa, using Supplementary Equation (46) at $r = 100$ μm, we get $c_{\text{min}} = 3.5 \times 10^{11}$ molec cm^{-3} which is equal to 12.5 ppb. The concentration of CO_2 around leaf cells investigated in [20] is of the order of 100 ppm. Therefore, our sensor can be expected to be sensitive enough to measure CO_2 exchange of photosynthetic cells with a high resolution.

SUPPLEMENTARY NOTE 9. MEASUREMENT OF THE ACOUSTIC WAVES GENERATED BY THE NANOSCALE VIBRATIONS OF CELLS OR BACTERIA

Bio-identifications are required in various fields including medicine, food and beverages, water, safety, public health and security. Current procedures of bacteria detection are costly,

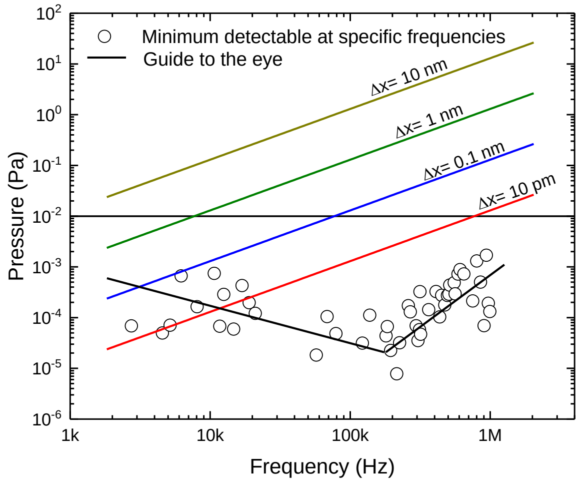

Supplementary Figure 9: Opto-mechanical acoustic sensor performance for cell vibration detection. The circles are experimental sensitivities (at a bandwidth of 1 Hz) for our sensor with given size. Our sensor is scalable and it can be fabricated slightly larger or smaller thereby changing its sensitivity at different frequency. However, more or less the solid black line shows a guide to the eye for expected sensitivity of such sensor we have. The colour solid lines show the amount of pressure generated by the cell versus frequency for different displacements Δx.

time-consuming and based on cell culture which require laboratory and microscopic analysis done by a trained person. However, self-contained mobile bio-sensors can simplify fast diagnosis in place even for the some bacteria that can not be cultured in laboratory [22].

There are recent experiments on bacteria, yeast and plant cell samples in liquid and soil which show bacteria and yeast produce vibrations with displacement amplitudes of 1-100 nm and plant cells produce vibrations with displacement amplitude of 1-30 nm [23–26]. These experiments are performed for cell concentrations of 108 and 107 CFU ml^{-1} which respectively include 96 and 27 bacteria per sample [25]. Our opto-mechanical acoustic sensor can be used to study micro-organisms through detecting these vibrations and hence there is no need to grow cells in an especial probing medium. We can consider a very thin layer of bacteria or cell in a liquid which is coated on a silicon or glass substrate and hold our acostic sensor very close to the sample such that we can ignore the air attenuation of the acoustic waves generated by cells. In order to estimate if our sensor is sensitive enough to probe such vibrations, we need to estimate the pressure produced by these micro-orgnisms vibrations and compare it with the minimum presure that our sensor can detect at a given

frequency. The pressure generated by the vibrational displacements can be calculated as

$$P = \pi \nu_0 Z_{\mathrm{air}} d, \tag{47}$$

in which ν_0 is the vibration frequency, Z_{air} =413 Pa s m^{-1} is air impedance and d is the displacement. For ν_0 = 10 kHz and d =1 nm we have $P = 1.3 \times 10^{-2}$ Pa which suggests that our acoustic sensor should be able to quite easily detect cell vibrations.

Supplementary Figure 9 shows how the opto-mechanical sensor performs for cells vibrating at other frequencies and with smaller displacement amplitudes. As can be seen, the sensor should be applicable to sensitively detect small cellular vibrations at frequencies across the full range over which it has been calibrated. We note, further, that the broadband sensitivity of better than 10 mPa Hz$^{-1/2}$ is sufficient to monitor cellular vibrations across the full continuous frequency range. One technical consideration is that, as with other resonant sensors and as shown in Supplementary Figure 7, the responsively of the sensor fluctuates significantly over the measurement band. These fluctuations are static in time, and therefore could be compensated for in post-processing to produce an accurate spectrum of cellular vibrations. Alternatively, as discussed in the main text and in more detail later in the Supplementary Note 9, optomechanical cooling techniques could be employed to broaden the mechanical resonances and flatten the response without degrading singnal-to-noise.

Our opto-mechanical acoustic sensor can also offer some other advantages for cell detection such as using this sensor one does not need an agent as in [23–26] since the sensor can measure cells vibration without need to attach them to the sensor. Moreover, we can detect the magnitude and frequency of the vibrations and scan over xy coordinates to map the vibrational pattern. This enables studying and investigating different bacteria in a sample. This sensing enables the experimenter to measure spectral density of the acoustic waves generated by the cell which may help to understand the difference between different types of cells (maybe cancer detection) or many other cell biology investigation such as probing fast phenomena happening on the cell wall or inside it. The cell wall can also behave like a membrane transferring internal oscillations to the air and finally to the sensor. This suggest that our sensor has significant potential in developing micromechanical sensors for micro-organisms.

SUPPLEMENTARY NOTE 10. OPTOMECHANICAL COOLING

As discussed in the main text, a range of techniques have been developed in the quantum optomechanics community to cool the motion of mechanical resonators (see e.g. [27–32]). These cooling processes also, by necessity broaden the mechanical resonances. This broadening can be used to flatten the resonant response of the sensor. Unlike other methods to broaden mechanical resonance, for example, by introducing additional damping, however, since the ideal laser acts as a zero temperature bath [15], these quantum optomechanical cooling techniques do not add additional thermal noise. Therefore, they can be used to flatten the response of an optomechanical system without the usual cost of additional noise. The fractional broadening of the mechanical resonances which is possible can be quantified using a single parameter, the optomechanical cooperativity

$$C = \frac{4g_0^2 N}{\kappa\gamma}, \tag{48}$$

where g_0 is the vacuum optomechanical coupling rate, N is the number of intracavity photons, κ is the optical decay rate, and γ is the mechanical resonance linewidth. Optomechanical cooperativities in the range of 10^3 to 10^6 can generally be readily achieved [33]. Application of, for example, feedback cooling [30, 31], can broaden the mechanical linewidth by as much as a factor of C. So, for example, a mechanical resonance at 500 kHz with a quality factor of 1,000 (and therefore linewidth of 500 Hz) could to broadened to give a near-flat response.

SUPPLEMENTARY NOTE 11. DEVICE FABRICATION PROCESS

Supplementary Figure 10 shows the process used to fabricate the ultrasound sensor.

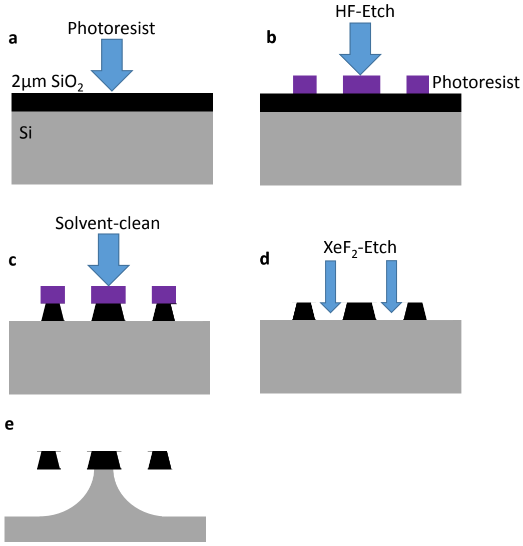

Supplementary Figure 10: Cross-sectional view of the microfabrication process. a)-c):Starting from a Oxide-coated Silicon wafer, UV-photolithography and HF-wet-etch were performed to define the Silica structure. d) and e): A XeF_2 dry-etch isotropically removes silicon and releases the silica-structure.

SUPPLEMENTARY REFERENCES

[1] Kim, K. H. et al. Air-coupled ultrasound detection using capillary-based optical ring resonators. *Sci. Rep.* **7,** 109 (2017).

[2] Kuntzman, M. L., & Hall, N. A. A broadband, capacitive, surface-micromachined, omnidirectional microphone with more than 200 kHz bandwidth. *J. Acoust. Soc. Am.* **135,** 3416-3424 (2014).

[3] Jo, W., Akkaya, O. C., Solgaard, O., & Digonnet, M. J. Miniature fiber acoustic sensors using a photonic-crystal membrane. *Opt. Fiber Technol.* **19,** 785-792 (2013).

[4] Hansen, S. T., Ergun, A. S., Liou, W., Auld, B. A., & Khuri-Yakub, B. T. Wideband micromachined capacitive microphones with radio frequency detection. *J. Acoust. Soc. Am.* **116,** 828-842 (2004).

[5] Bucaro, J. A., Lagakos, N., Houston, B. H., Jarzynski, J., & Zalalutdinov, M. Miniature, High performance, low-cost fiber optic microphone. *J. Acoust. Soc. Am.* **118,** 1406-1413 (2005).

[6] Jo, W., Kilic, O., & Digonnet, M. J. Highly sensitive phase-front-modulation fiber acoustic sensor. *J. Light. Technol.* **33,** 4377-4383 (2015).

[7] Akkaya, O. C., Akkaya, O., Digonnet, M. J., Kino, G. S., & Solgaard, O. Modeling and Demonstration of Thermally Stable High-Sensitivity Reproducible Acoustic Sensors. *J. Microelectromech. Syst.* **21,** 1347-1356 (2012).

[8] Ma, J. et al. Fiber-Optic FabryPerot Acoustic Sensor With Multilayer Graphene Diaphragm. *IEEE Photon. Technol. Lett.* **25,** 932-935 (2013).

[9] Kilic, O., Digonnet, M., Kino, G., & Solgaard, O. External fibre FabryPerot acoustic sensor based on a photonic-crystal mirror. *Meas. Sci. Technol.* **18,** 3049 (2007).

[10] Martin, D. T. et al. A Micromachined Dual-Backplate Capacitive Microphone for Aeroacoustic Measurements. *J. Microelectromech. Syst.* **16,** 1289-1302 (2007).

[11] Bucaro, J. A., & Lagakos, N. Lightweight fiber optic microphones and accelerometers. *Rev. Sci. Instrum.* **72,** 2816-2821 (2001).

[12] Preisser, S. All-optical highly sensitive akinetic sensor for ultrasound detection and photoacoustic imaging. *Biomed. Opt. Express*, **7,** 4171-4186 (2016).

[13] Gardiner, C. & Zoller, P. *Quantum Noise: A Handbook of Markovian and Non-Markovian Quantum Stochastic Methods With Applications to Quantum Optics 3rd edn.,* (Springer Science & Business Media, 2004).

[14] Walls, D.F. & Milburn, G.J. *Quantum Optics 2nd edn.,* (Springer Science & Business Media, 2007).

[15] Bowen, W.P. & Milburn, G.J. *Quantum Optomechanics 1st edn.,* (CRC Press, 2015).

[16] Bass, H. E., Sutherland, L. C., & Zuckerwar, A. J. Atmospheric absorption of sound: Update. *J. Acoust. Soc. Am.*, **88,** 2019-2021 (1990).

[17] Bao, M. *Analysis and design principles of MEMS devices 1st edn.,* (Elsevier, 2005).

[18] Anetsberger, G., Rivière, R., Schliesser, A., Arcizet, O., & Kippenberg, T. J. Ultralow-dissipation optomechanical resonators on a chip. *Nat. Photon.* **2,** 627-633 (2008).

[19] Arcizet, O., Riviere, R., Schliesser, A., Anetsberger, G., & Kippenberg, T. J. Cryogenic properties of optomechanical silica microcavities. *Phys. Rev. A*, **80,** 021803 (2009).

[20] Ho, Q. T., Verboven, P., Yin, X., Struik, P. C., & Nicolaï, B. M. A Microscale Model for Combined CO2 Diffusion and Photosynthesis in Leaves. *PloS One*, **7,** e48376 (2012).

[21] Tam, A. C. Applications of photoacoustic sensing techniques. *Rev. Mod. Phys.* **58,** 381 (1986).

[22] Ahmed, A. et al. Biosensors for Whole-Cell Bacterial Detection. *Clin. Microbiol. Rev.* **27,** 631-646 (2014).

[23] Longo, G. Rapid detection of bacterial resistance to antibiotics using AFM cantilevers as nanomechanical sensors. *Nat. Nanotechnol.* **8,** 522-526 (2013).

[24] Kasas, S. et al. Detecting nanoscale vibrations as signature of life. *Proc. Natl. Acad. Sci. U.S.A.* **112,** 378-381 (2015).

[25] Lissandrello, C. et al. Nanomechanical motion of Escherichia coli adhered to a surface. *Appl. Phys. Lett.* **105,** 113701 (2014).

[26] Song, L. et al. Nanoscopic Vibrations of Bacteria with Different Cell-Wall Properties Adhering to Surfaces under Flow and Static Conditions. *ACS nano*, **8,** 8457-8467 (2014).

[27] Chan, J. et al. Laser cooling of a nanomechanical oscillator into its quantum ground state. *Nature*, **478,** 89-92 (2011).

[28] Teufel, J. D. et al. Sideband cooling of micromechanical motion to the quantum ground state. *Nature*, **475,** 359-363 (2011).

[29] Schliesser, A. et al. Resolved-sideband cooling of a micromechanical oscillator. *Nat. Phys.* **4,** 415-419 (2008).

[30] Lee, K. H., McRae, T. G., Harris, G. I., Knittel, J., & Bowen, W. P. Cooling and Control of a Cavity Optoelectromechanical System. *Phys. Rev. Lett.* **104,** 123604 (2010).

[31] Harris, G.I., Andersen, U.L., Knittel, J., & Bowen, W. P. Feedback-enhanced sensitivity in optomechanics: Surpassing the parametric instability barrier. *Phys. Rev. A*, **85,** 061802(R) (2012).

[32] Kim, P.H., Hauer, B.D., Clark, T.J., Fani Sani, F., Freeman, M.R., Davis, J.P. Magnetic actuation and feedback cooling of a cavity optomechanical torque sensor. *Nat. Commun.* **8,** 1355 (2017).

[33] Aspelmeyer, M., Kippenberg, T. J., & Marquardt, F. Cavity optomechanics. *Rev. Mod. Phys.* **86,** 1391 (2014).
